# Total Thrombus-Formation Analysis System (T-TAS) in Aortopathies: A Conceptual and Potential Framework to Spatial Heterogeneity and Regional Context

**DOI:** 10.3390/ijms27073144

**Published:** 2026-03-30

**Authors:** Sebastian Krych, Julia Gniewek, Marek Kolbowicz, Marta Stępień-Słodkowska, Maria Adamczyk, Tomasz Hrapkowicz, Paweł Kowalczyk

**Affiliations:** 1 Department of Cardiac, Vascular and Endovascular Surgery and Transplantology, School of Medical Sciences in Zabrze, Medical University of Silesia, Marii Skłodowskiej-Curie 9, 41-800 Zabrze, Poland; thrapkowicz@sum.edu.pl; 2 Student’s Scientific Society, Department of Cardiac, Vascular and Endovascular Surgery and Transplantology, School of Medical Sciences in Zabrze, Medical University of Silesia, Marii Skłodowskiej-Curie 9, 41-800 Zabrze, Poland; julia.m.gniewek@gmail.com; 3 Institute of Physical Culture Sciences, University of Szczecin, Piastow 40b/6, 71-065 Szczecin, Poland; marek.kolbowicz@usz.edu.pl (M.K.); marta.stepien-slodkowska@usz.edu.pl (M.S.-S.); 4 Institute of Spatial Management and Socio-Economic Geography, University of Szczecin, Mickiewicza 64, 71-101 Szczecin, Poland; maria.adamczyk@usz.edu.pl; 5 Department of Animal Nutrition, The Kielanowski Institute of Animal Physiology and Nutrition, Polish Academy of Sciences, Instytucka 3, 05-100 Jabłonna, Poland

**Keywords:** Total Thrombus-Formation Analysis System (T-TAS), aortopathy, hemostasis, thrombogenesis, haemodynamics, arterial wall mechanics, microfluidics thrombosis assays, von Willebrand factor (vWF)–ADAMTS13 axis, two-way fluid–structure interaction (FSI), spatial mangement, patient-specific haemodynamics, computational modelling

## Abstract

Thoracic aortopathies, including aneurysm and dissection, are complex vascular disorders characterized by structural alterations of the aortic wall that disrupt normal haemodynamics. Altered shear stress, turbulent flow, and endothelial dysfunction promote thrombus formation and modulate systemic hemostasis via platelet activation and the von Willebrand factor–ADAMTS13 axis. The Total Thrombus-Formation Analysis System (T-TAS) is a microfluidic, flow-dependent assay that quantitatively evaluates thrombus formation under physiological shear conditions. Although studied in various cardiovascular contexts, its application in aortopathies remains largely unexplored, and no prospective studies have validated its clinical utility. Integrating T-TAS with computational haemodynamic approaches, such as two-way fluid–structure interaction simulations, enables assessment of the interplay between blood flow, vessel wall mechanics, pulse wave propagation, and local shear patterns. Patient-specific modelling, including individualized flow profiles, pressure distributions, and wall properties, may enhance mechanistic insights. Genetic variants in Fibrillin-1 gene (FBN1), Transforming Growth Factor Beta Receptor 1/2 (TGFBR1/2), Actin Alpha 2 (ACTA 2), and Myosin Heavy Chain 11 (MYH11) further contribute to structural vascular heterogeneity and diverse systemic haemostatic phenotypes, highlighting the need for personalized assessment. T-TAS should currently be considered an exploratory research tool rather than a validated diagnostic or prognostic method. This narrative review proposes a hypothesis-generating framework integrating structural, haemodynamic, molecular, and functional perspectives. Combining flow-based thrombosis assays with advanced modelling may inform future translational studies, improve mechanistic understanding of thrombus formation, and support personalized risk stratification and management in patients with thoracic aortopathies.

## 1. Introduction

Aortopathies constitute a heterogeneous group of disorders involving structural and functional abnormalities of the aortic wall, including aneurysmal degeneration, dissection, and genetically determined aortic disease. These conditions are associated with altered blood flow patterns, endothelial injury, and disturbances of hemostasis, which together predispose patients to both thrombotic and bleeding complications [1].

Aortopathy encompasses a wide spectrum of pathological changes, such as aneurysm formation, dissection, and non-aneurysmal mural abnormalities. These conditions frequently disrupt normal blood flow dynamics and damage the endothelial surface, creating a prothrombotic environment that can promote intraluminal thrombus formation or mural thrombi [2]. Mural or floating thrombi in the aorta, although relatively uncommon, have significant clinical implications, including systemic embolism, acute limb ischemia, and increased morbidity and mortality [3].

The pathophysiology of thrombosis in aortopathy is closely linked to mechanical and hemodynamic disturbances within the aortic wall. Diseases such as aortic dissection, aneurysms, and acute aortic syndromes demonstrate how structural disruption and turbulent blood flow contribute to thrombus formation, consistent with the principles of Virchow’s triad, which identifies vascular wall injury, abnormal blood flow, and alterations in blood constituents as key drivers of thrombogenesis [4,5].

## 2. The Total Thrombus-Formation Analysis System

### 2.1. Principles of the Total Thrombus-Formation Analysis System

The Total Thrombus-Formation Analysis System (T-TAS) is a novel laboratory tool that enables dynamic assessment of hemostasis under conditions approximating physiological blood flow. It evaluates thrombus formation in whole blood passing through microchannels under shear stress, simultaneously assessing platelet function and coagulation cascade activity. This differentiates T-TAS from conventional static hemostatic assays activated partial thromboplastin time (APTT), platelet chip (PT) and platelet aggregometry, which typically evaluate only isolated aspects of primary or secondary hemostasis. Literature evidence indicates that T-TAS can detect subtle platelet dysfunctions and coagulation abnormalities that are not apparent in standard laboratory tests, highlighting its potential utility for assessing bleeding and thrombotic risks in various patient populations, including those receiving anticoagulant or antiplatelet therapy [6,7,8,9,10,11,12,13,14].

The T-TAS was developed to address this gap by enabling real-time assessment of thrombus formation in whole blood under controlled shear stress (refers to the tangential force exerted by flowing blood on the vascular endothelium, which plays a key role in platelet activation, von Willebrand factor conformational changes, and shear-dependent thrombus formation). By integrating platelet function and coagulation activity, T-TAS may be particularly relevant in vascular diseases where hemodynamics plays a central role, such as aortopathies [6]. T-TAS is a microchip-based flow chamber system that evaluates thrombus formation by perfusing citrated whole blood through microchannels coated with thrombogenic substrates. Two main microchips are used: the platelet (PL) chip, coated with collagen, and the atheroma chip (AR), coated with collagen and tissue factor. Blood is perfused at predefined flow rates, generating arterial or venous shear stress conditions [7]. Thrombus formation gradually occludes the microchannels, leading to an increase in flow pressure which is continuously recorded. Quantitative parameters, such as time to occlusion and area under the pressure–time curve, reflect the overall thrombogenic potential of the blood sample. Unlike traditional platelet aggregometry, T-TAS preserves cellular interactions and flow dynamics, providing a more physiologically relevant assessment of hemostasis [8]. According to a scoping review of the T-TAS literature, the system has been studied extensively in cardiovascular settings—including bleeding disorders, antiplatelet/anticoagulant monitoring, and general thrombotic risk assessment—although its application in specific large-artery diseases remains limited [9].

#### 2.1.1. Functional Assessment of Hemostasis in Aortopathies: Advantages and Limitations of T-TAS

Sustained exposure of circulating blood elements to such altered flow environments may induce systemic adaptations in their functional phenotype, potentially detectable in peripheral blood as an altered global hemostatic profile rather than a localized vascular defect [7,8].

##### Advantages of T-TAS

T-TAS offers several important advantages over conventional hemostatic assays:

- Flow-based, physiologically relevant assessment: Unlike standard coagulation tests such as prothrombin time (PT) or activated (APTT), which are performed under static conditions, T-TAS evaluates thrombus formation under controlled flow and shear stress, better approximating in vivo arterial conditions [1,2,3].
- Integrated analysis of hemostasis: The system simultaneously captures platelet adhesion, aggregation, and coagulation processes within a single assay, reflecting the dynamic interplay between cellular and plasmatic components of hemostasis [1,2].
- Whole-blood evaluation: By using whole blood, T-TAS measures the net thrombogenic or hemorrhagic potential arising from interactions among platelets, coagulation factors, and other circulating elements [2,6].
- Detection of global hemostatic phenotype: T-TAS enables identification of an overall prothrombotic or hypocoagulable state as an integrated functional outcome, rather than isolated pathway abnormalities [6,7].
- Complementary role in multimodal assessment: Functional data obtained from T-TAS may be correlated with imaging findings (e.g., aortic diameter, dissection, wall remodeling) and circulating biomarkers such as transforming growth factor-β (TGF-β), von Willebrand factor (vWF), and markers of endothelial activation, supporting a more comprehensive understanding of disease mechanisms [3,4,5,6,7,8].
- Exploratory and potential: The system may facilitate translational research aimed at linking structural vascular abnormalities with systemic hemostatic consequences, particularly in the context of chronic hemodynamic stress [6,7,8,9].

##### Limitations of T-TAS

Despite its advantages, T-TAS has several important limitations:

- Lack of structural assessment: The system does not provide any information on aortic morphology, vessel wall integrity, or localization of pathology, and cannot detect aneurysms, dissections, or endothelial damage.
- No anatomical specificity: T-TAS reflects systemic hemostatic behavior and does not allow identification of the vascular source of abnormalities.
- Limited mechanistic insight: While it captures the net effect of thrombus formation, it does not differentiate specific pathways of platelet activation or coagulation factor deficiencies, and therefore, does not replace targeted diagnostic assays [8].
- Standardized rather than patient-specific conditions: The assay does not model individual aortic biomechanics or patient-specific flow characteristics, in contrast to advanced computational simulations.
- Unproven clinical relevance in aortopathies: Currently, there is no definitive clinical evidence demonstrating persistent systemic hemostatic remodeling in patients with aortic disease. The concept that chronic hemodynamic disturbances lead to measurable, reproducible alterations in thrombus formation remains hypothetical and requires validation in prospective studies.
- Indirect assessment of endothelial function: T-TAS does not directly evaluate endothelial dysfunction but instead reflects the integrated response of circulating blood elements exposed to prior vascular conditions.

Aortopathies remain diseases of primarily structural origin requiring imaging-based and genetic diagnostics. The role of T-TAS is best understood within a multimodal framework integrating structural, molecular, and functional data, and it currently serves primarily as a modality rather than a standalone diagnostic test [1,2,3,4,5,6,7,8,9,10].

The major advantages of T-TAS include whole-blood analysis, incorporation of flow conditions, and rapid turnaround time. However, limitations include the absence of an endothelial component, limited standardization across centers, and scarce data specific to aortopathies. Further validation is required before routine clinical implementation in this patient population [9]. Despite strong biological plausibility, there are currently no robust clinical trials or large cohort studies directly evaluating T-TAS metrics in patients with aortopathy. Available research on T-TAS focuses largely on coronary artery disease, atrial fibrillation, and antithrombotic therapy monitoring [9].

#### 2.1.2. Rationale for Linking Aortopathy to T-TAS

The rationale for applying T-TAS in the context of aortopathy arises from three core considerations:

*(a) Aortopathy Alters Thrombogenic Potential*

Structural abnormalities of the aorta—such as aneurysmal dilations or dissection flaps—generate regions of disturbed flow and endothelial dysfunction that favor platelet activation and coagulation pathways. Although this is not a direct result of the T-TAS study, this concept is supported by models showing that altered hemodynamics in aortic diseases predispose to clot formation [10,11,12].

*(b) T-TAS Quantifies Flow-Dependent Thrombus Formation*

T-TAS provides a distinct methodological perspective in that it measures thrombus growth under physiologic shear conditions, capturing platelet and coagulation contributions to thrombus formation in whole blood samples. Its use has been established in cardiovascular disease populations for evaluating bleeding risk and antithrombotic therapy efficacy. Although the literature on T-TAS does not yet include large prospective studies on aortic disease cohorts, the technology’s sensitivity to platelet-dependent thrombogenicity under flow makes it a promising candidate for future research in large vessel pathologies [6,7,8,9,10,11,12,13,14].

*(c) Potential Research and Clinical Application*

The successful application of T-TAS in other vascular conditions suggests it could be useful in characterizing thrombotic risk in aortopathy, including:

- Assessing whether patients with aortic aneurysms or dissection have distinct thrombogenic profiles;
- Monitoring changes in thrombogenicity following interventions (e.g., surgical repair, endovascular therapy);
- Guiding individualized antithrombotic therapy in patients with significant aortic pathology.

**Figure 1:**
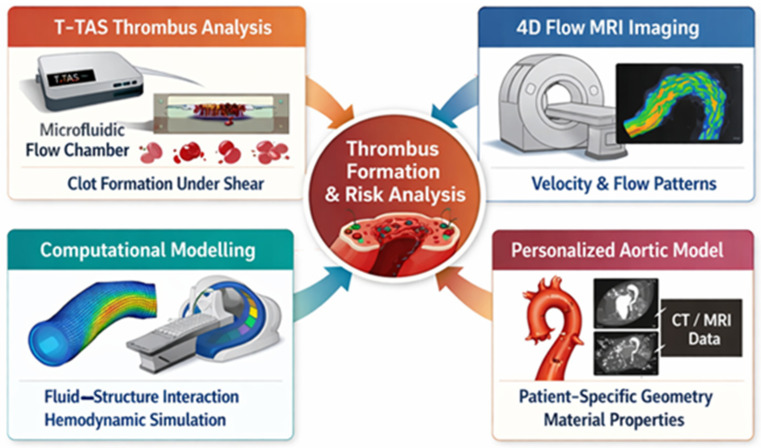
Integration of T-TAS, Hemodynamics and Patient-Specific Aortic Modelling.

To date, most evidence on aortic thrombus formation comes from case reports and descriptive studies. For instance, spontaneous aortic thrombus findings often relate to underlying pathology or systemic prothrombotic states, emphasizing the need for refined analytical tools [15,16] (Figure 1).

## 3. Potential Applications of T-TAS in Aortic Disease

### 3.1. Established Clinical Applications of T-TAS

Although the utility of T-TAS in specific cardiac surgical populations remains under investigation, there is an emerging body of clinical evidence demonstrating its applicability in various cardiovascular disease settings and its potential to inform hemostatic risk beyond experimental assumptions. T-TAS has been investigated in several cardiovascular contexts, including coronary artery disease, antiplatelet therapy monitoring, and device-related thrombogenicity. These studies demonstrate sensitivity to flow-dependent hemostatic changes; however, direct clinical evidence in aortopathies remains limited [6,17,18]. In a large cohort of over 500 CAD patients undergoing coronary angiography, Area Under the Curve 30 (AR10-AUC30) levels measured by T-TAS were significantly lower in individuals who experienced 1-year bleeding events, and low AR10-AUC30 was independently associated with bleeding risk, supporting its prognostic value in clinical practice [6,17,18].

Similarly, in patients with atrial fibrillation undergoing catheter ablation, (AR10-AUC30) measured on the day of the procedure predicted periprocedural bleeding events, suggesting that T-TAS may contribute to individualized procedural risk stratification [6,17,18]. Beyond thrombotic and bleeding risk prediction in atherosclerotic and arrhythmia populations, T-TAS has demonstrated utility in pediatric Fontan patients, where both AR and PL AUC values were lower compared with controls and significantly modified by antithrombotic therapies, indicating its potential role in monitoring thrombogenicity in complex congenital heart disease [6,10,11,12,13,14,15,16,17,18]. Additionally, in patients supported with continuous-flow left ventricular assist devices (CF-LVADs), T-TAS parameters revealed severely impaired platelet thrombus-formation capacity consistent with acquired von Willebrand syndrome, and differences in PL24-AUC10 and AR10-AUC30 levels correlated with device-related hemostatic alterations, highlighting its usefulness in detecting clinically relevant hemostatic dysfunction associated with mechanical circulatory support [6,8,9,10,11,12,13,14,15,16,17,18,19,20,21,22].

A recent scoping review of the literature confirms that T-TAS has been applied to diverse clinical situations—including bleeding disorders, monitoring of anticoagulant and antiplatelet therapy, and prediction of bleeding risk—underscoring its broader relevance in thrombotic and hemorrhagic risk assessment across cardiovascular populations [8,10]. Taken together, these studies provide real clinical evidence that T-TAS offers functional information in vivo that complements conventional platelet assays and coagulation tests, and they support its further evaluation in cardiac surgical and interventional settings.

#### 3.1.1. Cardiovascular Disease and Antithrombotic Therapy

T-TAS has been extensively studied in patients with coronary artery disease and those receiving antiplatelet or anticoagulant therapy. Reduced thrombus formation parameters on the PL chip have been associated with an increased risk of bleeding, while Atheroma chip (AR chip) measurements correlate with the intensity of anticoagulation. These findings support the use of T-TAS as a global assay for balancing thrombotic and hemorrhagic risk [6,13,14,15,16,17,18].

#### 3.1.2. Bleeding Disorders and Platelet Dysfunction

In congenital bleeding disorders, including von Willebrand disease, T-TAS has demonstrated sensitivity to defects in platelet adhesion and aggregation, particularly under high shear stress conditions. However, its ability to detect mild platelet dysfunction remains limited, and results should be interpreted in conjunction with established laboratory assays [19,20,21,22,23,24,25,26,27,28,29,30,31,32].

#### 3.1.3. Aortic Aneurysm and Dissection

Although direct clinical studies are limited, the pathophysiology of aortic aneurysm and dissection suggests potential applicability of T-TAS. Low shear stress regions within aneurysms favor fibrin-rich thrombus formation, whereas high shear stress near intimal tears promotes platelet activation. T-TAS could theoretically differentiate these prothrombotic states and help stratify risk [21].

#### 3.1.4. Aortic Valve and Endovascular Interventions

Studies in patients undergoing transcatheter aortic valve implantation (TAVI) have demonstrated significant changes in T-TAS-derived thrombogenicity indices following the procedure, reflecting altered shear stress and acquired von Willebrand factor abnormalities. These findings support the concept that T-TAS is sensitive to hemodynamic changes relevant to aortic pathology [22].

#### 3.1.5. Assessment of Bleeding and Thrombotic Risk

Patients with aortopathies, particularly genetically mediated forms such as Marfan syndrome, Loeys–Dietz syndrome, or familial thoracic aortic aneurysm and dissection (FTAAD), exhibit structural weakening of the aortic wall, predisposing them to dissection, aneurysm formation, and other vascular complications [33,34,35,36,37]. In these settings, impaired vascular integrity, altered wall mechanics, and disturbed flow patterns may promote local platelet activation and coagulation or, conversely, increase the risk of bleeding during surgical or invasive procedures [38,39,40]. Conventional laboratory tests frequently fail to fully capture this complex hemostatic environment, particularly regarding interactions between the vessel wall and circulating hemostatic elements. T-TAS measures parameters such as Occlusion Start Time (OST), Occlusion Time (OT), and (AUC), which reflect the global thrombus-forming capacity of whole blood [8,41,42,43,44,45]. Low AUC values, particularly on the AR chip (assessing both primary and secondary hemostasis), have been demonstrated to predict subsequent bleeding events in patients with cardiovascular disease undergoing invasive procedures, including percutaneous coronary interventions and atrial fibrillation ablation, and in those receiving anticoagulant therapy. Thus, T-TAS application in aortopathy patients may provide additional prognostic information regarding hemostatic complications—both excessive thrombosis leading to microvascular emboli and bleeding tendencies—thereby directly influencing clinical decision-making for surgical and pharmacologic management [8,46,47,48,49,50,51].

#### 3.1.6. Monitoring Anticoagulant and Antiplatelet Therapy

Management of patients with aortopathies frequently includes prophylactic anticoagulation or antiplatelet therapy, particularly following surgical interventions or in the presence of additional thromboembolic risk factors [6,7,8,9,10,11,12,13,14]. However, responses to therapy may vary: some patients remain in a prothrombotic state despite treatment, whereas others experience bleeding despite nominally “therapeutic” laboratory values. In such scenarios, reliance solely on conventional tests may yield misleading conclusions, as these assays do not simultaneously reflect the full spectrum of hemostatic activity under flow conditions. T-TAS, by evaluating thrombus formation in vitro under flow conditions, may better reflect the impact of anticoagulant or antiplatelet therapy on platelet activity and the coagulation cascade in whole blood, making it a promising tool for individualized therapy. It may also facilitate monitoring of patient preparation prior to aortic surgery, titration of anticoagulant dosing, and assessment of thrombotic or bleeding risk in the context of their hemostatic profile [6,7,8,9,10,11,12,13,14].

#### 3.1.7. Correlation with Genetic Phenotypes

A particularly promising application of T-TAS is in studies linking hemostatic profiles to specific genetic variants in patients with aortopathies. Mutations in genes involved in vascular structural integrity and cellular signaling—such as FBN1 (fibrillin-1), TGFBR1/TGFBR2 (TGF-β receptors), ACTA2 (smooth muscle α-actin), and MYH11 (smooth muscle myosin heavy chain)—are well-established risk factors for thoracic aortic aneurysms and dissections [47,48,49,50,51,52,53,54,55,56,57,58]. Knowledge of these genetic variants not only enables risk stratification for aortic disease progression and surgical decision-making but also suggests potential modifications in aortic wall mechanics with secondary implications for local hemostasis. For instance, FBN1 mutations result in fibrillin-1 dysfunction, weakening elastin microarchitecture and altering vessel wall interactions with blood flow and hemostatic elements, potentially affecting local platelet activation and thrombus formation [47,48,49,50,51,52,53,54,55,56,57,58]. Similarly, mutations in TGFBR1/TGFBR2 disrupt TGF-β signaling, modifying vascular remodeling and the balance between pro- and anti-thrombotic signals in the endothelium and vascular smooth muscle cells. Correlative studies employing T-TAS may identify hemostatic phenotypes associated with specific genetic variants, improving understanding of which patients are most susceptible to bleeding or thrombotic complications. This “genotype–phenotype–hemostasis” approach may inform personalized therapeutic strategies in both medical prophylaxis and surgical planning [47,48,49,50,51,52,53,54,55,56,57,58].

#### 3.1.8. Genetic Architecture of Aortopathies and Its Integration with Functional Hemostatic Assessment

Heritable thoracic aortic disease (HTAD), including thoracic aortic aneurysms (TAAs) and dissections, represents a genetically heterogeneous group of conditions in which pathogenic variants in specific genes profoundly influence disease onset, progression, and clinical outcomes. Recent large-scale cohort studies emphasize that gene-specific differences drive both prevalence and age of arterial events in syndromes such as vascular Ehlers–Danlos syndrome (COL3A1), Loeys–Dietz syndrome (TGFBR1/2), SMAD family transcription factors (SMAD3), TGFB2/3, and Marfan syndrome (FBN1), with significant implications for personalized surveillance strategies [47,58,59,60,61,62,63,64,65,66,67,68,69,70,71]. These findings underscore the necessity of genotype-tailored risk stratification for patients with HTAD, as gene and sex differences markedly alter the relative risk of aortic and arterial complications [47,58,59,60,61,62,63,64,65,66,67,68,69,70,71].

Inherited aortopathy is also increasingly studied outside syndromic contexts. Systematic genetic testing protocols identify pathogenic or likely pathogenic mutations in a substantial proportion of patients with sporadic thoracic aortic aneurysms and their first-degree relatives, with frequent involvement of genes such as *FBN1*, *TGFBR1/2*, *SMAD3*, and *COL3A1* [47,58,59,60,61,62,63,64,65,66,67,68,69,70,71]. However, despite extensive gene discovery efforts, a significant proportion of familial and sporadic cases remain genetically unexplained, highlighting the complex, polygenic architecture of aortopathy and the limitations of monogenic genotype–phenotype correlates alone.

Beyond canonical gene mutations, epigenetic and multiomic mechanisms are emerging as additional layers of genetic regulation in aortic disease. Recent work integrating DNA methylation and transcriptomic profiling in type A aortic dissection identified differentially methylated loci and gene expression signatures that may serve as novel diagnostic markers and mechanistic drivers of dissection pathogenesis [47,48,49,50,51,52,53,54,55,56,57,58,68,69,70,71]. Complementary reviews indicate that epigenetic modifications—including DNA methylation, histone alterations, and non-coding RNA regulation—modulate vascular smooth muscle cell function, extracellular matrix integrity, and endothelial homeostasis, thereby influencing aortic wall stability and disease progression [47,58,59,60,61,62,63,64,65,66,67,68,69,70,71]. Such epigenetic markers may offer dynamic, potentially reversible targets for early detection and therapeutic intervention, in contrast to fixed germline variants [27,72,73,74].

Integrating genetic and functional data into a unified clinical paradigm remains a key research frontier. The rationale for combining molecular genetics with functional hemostatic assays, such as the (T-TAS), stems from the recognition that structural gene defects not only predispose to mechanical weakening of the aortic wall but also alter downstream cellular and systemic responses, including endothelial function, shear sensitivity, and platelet–coagulation interactions. While T-TAS quantifies dynamic thrombus formation in whole blood under controlled flow conditions and does not replace imaging or genetic diagnostics, its parameters (e.g., AR_10_-AUC_30_ and PL-AUC) may capture combined effects of genotype-driven endothelial perturbations and hemodynamic stress on systemic hemostasis [7,8,9,47,48,49,50,51,52,53,54,55,56,57,58]. In this framework, specific genetic variants associated with HTAD could be correlated with distinct functional hemostatic profiles, enabling stratification of patients not only by structural risk but also by thrombogenic or bleeding propensity.

Importantly, such integrative approaches align with ongoing efforts to identify robust biomarkers that can bridge the gap between genotype and phenotype. Future translational studies should pursue multiomic profiling-including genomics, epigenomics, transcriptomics, and functional assays like T-TAS-to define signatures that more accurately predict clinical events such as dissection, rupture, or perioperative bleeding complications. This integrated strategy has the potential to refine risk prediction beyond aortic diameter and genetic predisposition alone, paving the way for precision medicine in aortic disease management also in athletes [8,75,76,77,78,79].

## 4. Hemodynamic Mechanisms Linking Aortopathy and Hemostasis

### 4.1. Hemostasis and Flow Disturbances in Aortopathies

**Figure 2:**
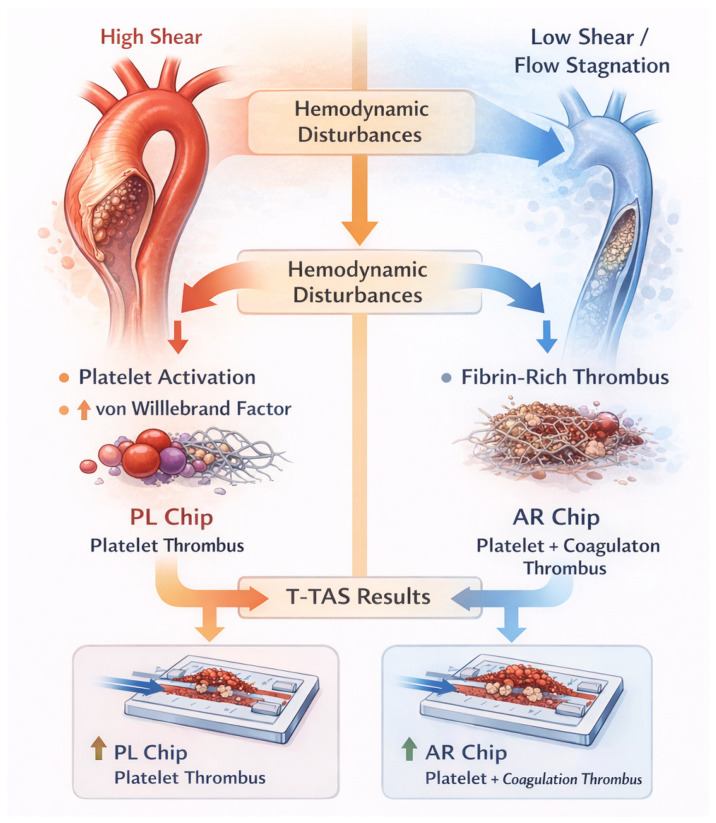
Shear Stress in the Aorta and T-TAS Readouts. Schematic representation of shear stress–dependent hemostatic alterations in aortopathies and their assessment using T-TAS. High shear stress regions (e.g., near intimal tears or stenotic segments) promote platelet activation and von Willebrand (vWF) factor multimer unfolding, whereas low shear stress or flow stagnation within aneurysmal sacs or false lumens favors fibrin-rich thrombus formation. T-TAS microfluidic chips simulate these flow conditions ex vivo, enabling integrated assessment of platelet-driven (PL chip) and platelet–coagulation–dependent (AR chip) thrombus formation.

**Figure 3:**
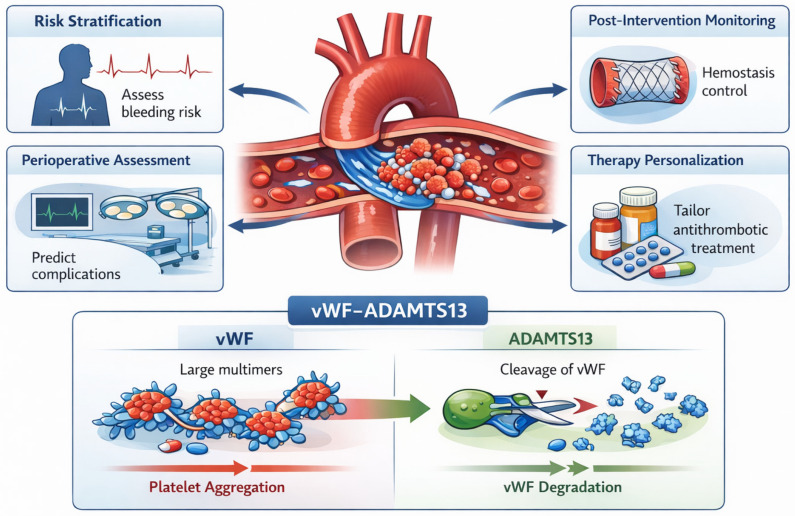
Functional interplay between aortic pathology, hemostasis, and the vWF–ADAMTS13 axis. The transverse vessel represents systemic circulation contributing to peripheral blood sampling. Schematic representation of the relationship between structural aortic disease, flow-dependent thrombus formation, and clinical applications of functional hemostatic assessment. Aortopathies are associated with altered hemodynamic conditions, including disturbed shear stress and flow turbulence, which promote platelet activation and thrombus formation within the aortic lumen. The horizontal channel intersecting the aorta represents a schematic depiction of intraluminal blood flow under shear conditions, rather than a distinct anatomical vessel. It is included to illustrate the site of flow-dependent thrombus formation and the interaction of circulating blood elements within a controlled flow environment. The lower panel illustrates the balance between von Willebrand factor (vWF) and ADAMTS13 activity. High-molecular-weight vWF multimers promote platelet adhesion and aggregation, whereas ADAMTS13 cleaves vWF into smaller, less thrombogenic forms, thereby regulating thrombus formation. The upper panels highlight potential clinical applications of flow-based hemostatic assessment, including risk stratification (bleeding vs. thrombotic risk), perioperative evaluation, post-intervention monitoring of hemostatic status, and personalization of antithrombotic therapy. This schematic emphasizes the complementary role of functional assays, such as flow-based thrombus formation analysis, in capturing the integrated hemostatic phenotype associated with aortic disease, alongside standard structural imaging and molecular diagnostics.

Aortopathies are characterized by complex alterations in blood flow, including regions of high shear stress near stenotic segments and low shear stress or flow stagnation within aneurysmal sacs or false lumens. These conditions influence platelet activation, von Willebrand factor cleavage, and fibrin formation. Clinical manifestations range from mural thrombus formation and distal embolization to bleeding complications following surgical or endovascular interventions. Standard coagulation tests fail to capture these flow-dependent phenomena. Therefore, functional assays that incorporate shear stress, such as T-TAS, may offer additional insight into the hemostatic status of patients with aortic disease [20] (Table 1, Figure 2 and Figure 3).

#### Comparison with PFA-100/200

The PFA-100/200 system represents another shear-dependent assay used clinically for screening platelet dysfunction and von Willebrand disease [1,2,3,4,5,6,7,8,9,10,11,12,13,14,15,16,17,18,19,20,21,22,23,24]. The assay measures closure time under high shear conditions in a capillary-based system coated with platelet agonists. Unlike T-TAS, which generates dynamic pressure–time curves and differentiates between platelet-dominant (PL chip) and platelet–coagulation–dependent (AR chip) thrombus formation [6,7,8,9,21], PFA primarily provides a single closure-time parameter. Both methodologies assess shear-dependent mechanisms; however, their analytical frameworks, output metrics, and levels of clinical validation differ [5,8,9]. T-TAS should therefore be considered one of several available functional flow-based assays rather than a standalone or superior alternative [9,76,80,81,82,83,84,85,86] (Figure 2 and Figure 3).

Overview of potential clinical applications of T-TAS in aortic disease. These include (i) thrombotic and bleeding risk stratification in patients with aortic aneurysm or dissection, (ii) perioperative hemostatic assessment in open or endovascular aortic surgery, (iii) monitoring of hemostatic changes following aortic valve or aortic interventions, and (iv) individualized optimization of antithrombotic therapy. The flow-based nature of T-TAS allows assessment of shear-dependent mechanisms not captured by conventional assays.

### 4.2. Distinctive Value of T-TAS in Aortic Disease

The Total Thrombus-Formation Analysis System (T-TAS) provides a distinct methodological perspective insights into hemostasis that are distinct from conventional coagulation and platelet function assays, providing a complementary perspective in the assessment of aortic disease where altered hemodynamics, shear stress, and endothelial dysfunction play a central role [1,4,10,11,12].

#### 4.2.1. Integration of Platelet Activity and Coagulation Pathways

- T-TAS evaluates thrombus formation in whole blood, capturing the interplay between platelets and coagulation factors under physiologic conditions [6,7,8,9,21].
- This contrasts with platelet aggregometry or isolated plasma-based assays, which assess components in isolation and may fail to reflect integrative, flow-dependent hemostatic interactions relevant to aortic pathology [5,6,7,8,9].

#### 4.2.2. Incorporation of Shear Stress and Dynamic Flow

Flow dynamics are central to aortic hemostasis, influencing platelet adhesion, aggregation, and von Willebrand factor (vWF)-mediated thrombus formation, particularly under conditions of disturbed or turbulent flow [10,11,20,21,22,23,24,25,26,27,28,29,30,31,32,33].

Standard coagulation tests (PT, aPTT) and thromboelastography (TEG/ROTEM) operate under near-static or low-shear conditions and do not replicate shear-dependent thrombus formation, limiting their sensitivity to flow-mediated dysfunction characteristic of aortic disease [5,6,7,8].

Thromboelastography (TEG) and rotational thromboelastometry (ROTEM) are clinically validated and widely implemented in cardiac surgery, trauma care, and perioperative medicine [5,8]. These modalities provide essential information regarding clot initiation, propagation, mechanical strength, and fibrinolysis under low-shear or near-static conditions [5]. They remain standard-of-care tools for global coagulation assessment in high-risk clinical scenarios. T-TAS should therefore be regarded as complementary rather than substitutive, offering a distinct flow-dependent perspective focused on shear-mediated platelet–coagulation interactions [5,6,7,8,9,10,11,12,13,14,15,16,17,18,19,20,21].

##### Comparison with Other Point-of-Care Hemostatic Assays

Other point-of-care assays, such as thromboelastography (TEG) and rotational thromboelastometry (ROTEM), provide valuable insights into coagulation dynamics and clot strength in various surgical and critical care settings [10,11]. However, these assays are largely static, performed under low-shear conditions, and primarily reflect global coagulation potential rather than flow-dependent platelet-vWF interactions. In contrast, T-TAS simulates physiologic shear and allows assessment of dynamic thrombus formation under controlled flow, which may capture hemostatic disturbances associated with aortopathy-induced turbulence or high-shear regions [4,5]. While this theoretical advantage is compelling, no studies to date have validated T-TAS against TEG/ROTEM specifically in aortic disease, representing a key gap for future research.

##### Integration with Genetic and Mechanistic Data

Integrating T-TAS with genetic profiling may provide further insights into how genotype-specific aortic pathologies influence systemic hemostasis. For example, patients with *FBN1* mutations may demonstrate early medial degeneration and altered endothelial function, which could theoretically affect shear-dependent thrombus formation. Similarly, variants in *TGFBR1/2* or *ACTA2* could modulate vascular smooth muscle cell response to stress, potentially influencing systemic coagulation profiles [1,2,3,12]. However, these connections remain conceptual, and no clinical trials have yet correlated T-TAS metrics with genotype, imaging findings, or clinical outcomes in aortopathy.

#### 4.2.3. Detection of Shear-Dependent Platelet Dysfunction

T-TAS is sensitive to abnormalities in the vWF–ADAMTS13 axis, capturing shear-dependent platelet dysfunction that is inadequately assessed by conventional assays [19,20,21,23,24,36,37,38].

This feature is particularly relevant in aortic disease, where pathological shear stress may induce vWF conformational changes, excessive proteolysis, or acquired von Willebrand syndrome, thereby amplifying both thrombotic and bleeding risk [25,26,27,28,29,30,32,33,34,40,41], (Table 2).

#### 4.2.4. Comparative Perspective and Complementarity

T-TAS does not replace thromboelastography, platelet aggregometry, or classical coagulation tests [5,8,9].

Rather, it complements these methodologies by providing a functional, flow-dependent readout that integrates molecular, cellular, and hemodynamic determinants of thrombus formation [6,7,8,9,21].

Together, these tools form a multi-modal framework for mechanistic research and hypothesis generation in aortic disease, highlighting the distinctive integrative value of T-TAS without implying diagnostic or prognostic superiority [4,8,9], (Table 2).

This table emphasizes methodological complementarity rather than diagnostic superiority of any single assay [5,8,9].

- T-TAS offers complementary information about a flow-dependent, integrative functional readout of thrombus formation, particularly relevant in aortic disease characterized by altered shear stress and disturbed hemodynamics [6,7,8,9,10,11,20,33].
- TEG/ROTEM, platelet aggregometry, and classical coagulation assays remain indispensable for evaluating baseline coagulation, clot mechanics, and isolated platelet function, offering complementary mechanistic insight [5,8].

**Figure 4:**
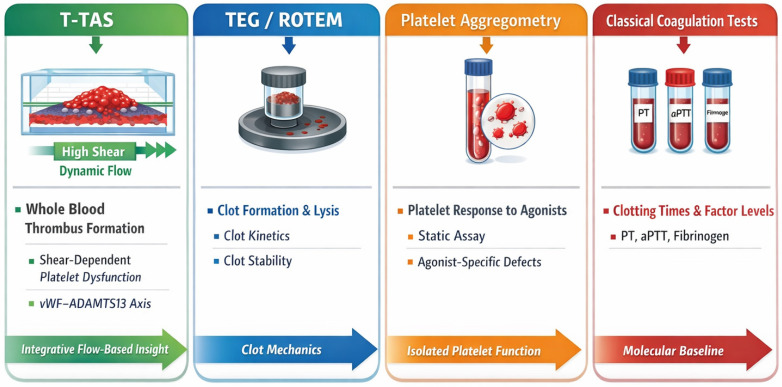
Comparative Hemostasis Assays in Aortic Disease (Complementary Insights into Hemostasis and Thrombosis).

To further illustrate the complementary nature of the hemostatic assays summarized in (Table 2 and Figure 4) provides a conceptual overview of how T-TAS, thromboelastography, platelet aggregometry, and classical coagulation tests interrogate distinct yet interconnected aspects of hemostasis and thrombosis in aortic disease.

#### 4.2.5. Conceptual Research Framework

The proposed research model is conceptual and has not yet undergone clinical validation. It is designed to explore whether chronic exposure to altered aortic hemodynamics is associated with measurable changes in systemic thrombus formation under flow conditions. The framework includes three principal components.

First, a comparative cross-sectional analysis would involve patients with genetically mediated aortopathies (e.g., Marfan syndrome, Loeys–Dietz syndrome), patients with degenerative thoracic aortic aneurysms, and healthy control subjects. Such stratification may allow assessment of whether distinct etiological backgrounds of aortic disease are associated with differences in integrated thrombus formation profiles under flow [1,2,3].

Second, T-TAS parameters would be correlated with quantitative hemodynamic metrics derived from advanced imaging modalities, including four-dimensional flow magnetic resonance imaging (4D-flow MRI) and shear stress indices [4,5]. Concurrent analysis of circulating biomarkers of endothelial activation—such as von Willebrand factor (vWF), soluble intercellular adhesion molecule-1 (sICAM-1), and endothelial-derived microparticles—would further enable investigation of potential associations between vascular wall activation and systemic hemostatic phenotype [6,7,8]. In addition, markers of platelet activation and thrombin generation could be assessed to explore their relationship with flow-dependent thrombus formation parameters [9,10].

Third, a longitudinal component would evaluate temporal changes in T-TAS profiles following surgical intervention or after hemodynamic normalization. Such analysis could help determine whether correction of abnormal aortic geometry and flow patterns is associated with measurable modulation of the integrated thrombotic phenotype [11,12,13].

The central hypothesis concerns the potential systemic functional correlates of local hemodynamic disturbances in aortic disease [10,11,20]. These may include shear-dependent alterations in vWF conformation and ADAMTS13-mediated cleavage, secondary platelet activation or exhaustion, and integrative changes in coagulation dynamics [23,24,25,33,40]. However, these relationships remain speculative in the context of aortopathies and require dedicated mechanistic and prospective validation studies [8,9].

These may include:

(I) Shear-dependent alterations in vWF conformation, affecting platelet adhesion and thrombus formation [20,21,22,23,24,33,40];

(II) Systemic platelet activation or exhaustion secondary to disturbed flow patterns [10,11,43].

Secondary changes in coagulation activation, reflecting conceptual hemostatic responses to altered vascular environments [3,4,12].

Accordingly, T-TAS is proposed not as a marker of local structural disease, but as a systemic, functional biomarker capturing thrombotic or bleeding tendencies arising from altered aortic flow environments [6,7,8,9,22,66]. This framing situates T-TAS as a complementary, exploratory tool for hypothesis generation and pathophysiological insight, rather than a standalone diagnostic or predictive assay.

##### Limitations and Practical Considerations of the Conceptual Framework

Several practical limitations restrict immediate clinical application of T-TAS in aortopathies. First, the assay requires fresh whole blood, which may be challenging in emergent settings such as acute type A dissection. Second, standardization across laboratories remains limited, and reproducibility can vary depending on chip type and flow conditions. Third, peripheral blood measurements may not fully reflect localized hemodynamic perturbations within the ascending or descending aorta, limiting spatial resolution. Finally, as noted above, all proposed applications are potential, and there is currently no evidence that T-TAS improves risk stratification, predicts clinical events, or guides therapy in aortic disease.

The primary pathology of aortic disease arises from structural alterations of the vessel wall rather than from changes in peripheral blood. Consequently, the T-TAS does not serve as a diagnostic tool for aortopathy, nor does it provide etiological insight into its development [6,7,8,9,21]. Nevertheless, T-TAS offers valuable functional information in this patient population. Specifically, it can: evaluate the systemic thrombotic phenotype [6,7,8,9,21] and assist in stratifying the risk of thrombotic or hemorrhagic complications [14,15,16,17,18,19,20,21,22,59,63,66].

Complement, but not replace, structural diagnostic modalities such as imaging and genetic testing [1,2,3,4,5,6,7,8,9,10,11,12,13,21,56,57,58,59].

In this context, its utility lies in functional phenotyping and risk assessment, providing information that may inform clinical management without implying causality for the structural vascular disease [9,21,43,65].

To date, the clinical applications of the T-TAS have predominantly focused on procedural contexts, including percutaneous coronary intervention (PCI), transcatheter (TAVI), coronary artery bypass grafting (CABG), and antiplatelet therapy monitoring [14,15,16,17,18,19,20,21,22,60,63,64,65,66]. While these studies demonstrate that T-TAS is sensitive to hemostatic changes induced by flow modification and vascular interventions, this does not automatically imply its utility in the assessment of aortopathies.

Rather, these findings highlight a potential research direction: T-TAS may capture functional alterations in thrombus formation associated with hemodynamic disturbances, but its application in aortic disease remains exploratory. Consequently, current evidence supports the use of T-TAS for functional phenotyping and risk stratification in procedural or pharmacologically modified settings, rather than as an established clinical tool for diagnosing or managing aortopathies [6,7,8,9,21,43,65].

### 4.3. Molecular Aspects of the vWF–ADAMTS13 Axis in Aortopathies and Its Relevance to T-TAS

Von Willebrand factor (vWF) and ADAMTS13 form a tightly regulated molecular axis that plays a central role in shear stress–dependent hemostasis. vWF is a large multimeric glycoprotein synthesized by endothelial cells and megakaryocytes and secreted into the circulation as ultra-large multimers (UL-vWFs), which exhibit high platelet-binding capacity. Under physiological conditions, the metalloprotease ADAMTS13 cleaves UL-vWFs into smaller, less prothrombotic multimers, thereby preventing excessive platelet aggregation [23,24].

#### 4.3.1. vWF–ADAMTS13 Dysregulation in Aortopathies

Aortopathies are characterized by profound hemodynamic disturbances, including regions of markedly increased shear stress (e.g., near intimal tears, stenotic segments, or prosthetic devices) and areas of low shear stress or flow stagnation (e.g., aneurysmal sacs or false lumens). High shear stress induces conformational unfolding of vWF multimers, exposing the A1 domain responsible for platelet glycoprotein Ib (GPIb) binding and rendering vWF susceptible to proteolytic cleavage by ADAMTS13. In conditions of sustained or excessive shear stress, as observed in advanced aortic disease or following aortic valve and endovascular interventions, accelerated cleavage of vWF may lead to acquired von Willebrand syndrome (AVWS), characterized by a loss of high-molecular-weight vWF multimers and an increased bleeding tendency. Conversely, regions of low shear stress and endothelial dysfunction may favor persistence of larger vWF multimers and fibrin-rich thrombus formation, contributing to mural thrombosis commonly observed in aortic aneurysms and dissections [25].

#### 4.3.2. Implications for T-TAS Readouts

The flow-based design of the Total Thrombus-Formation Analysis System (T-TAS) makes it particularly sensitive to disturbances in the vWF–ADAMTS13 axis. The PL chip, operating under high shear conditions and relying predominantly on platelet–vWF interactions, is well suited to detect reduced platelet thrombus formation associated with vWF depletion or dysfunction, as seen in AVWS. In contrast, the AR chip, which incorporates tissue factor and assesses combined platelet and coagulation pathway activation under lower shear conditions, may capture fibrin-dominant thrombus formation in low-flow environments typical of aneurysmal disease. Thus, T-TAS provides a functional, conceptual readout of shear-dependent molecular mechanisms involving vWF and ADAMTS13 that are not adequately assessed by conventional static assays. In the context of aortopathies, this capability highlights the potential of T-TAS as a complementary tool for evaluating the balance between bleeding and thrombosis driven by molecular and hemodynamic alterations [9,26].

#### 4.3.3. The vWF–ADAMTS13 Axis in TAVI, EVAR, and TEVAR: Implications for T-TAS Assessment

Transcatheter and endovascular aortic interventions, including (TAVI), endovascular (EVAR) and thoracic endovascular aortic repair (TEVAR), induce abrupt and profound changes in aortic geometry and blood flow patterns. These hemodynamic alterations directly affect the molecular balance between von Willebrand factor (vWF) and its cleaving protease ADAMTS13, thereby influencing peri- and post-procedural thrombotic and bleeding risk [27,28,29].

#### 4.3.4. TAVI: High Shear Stress and Acquired von Willebrand Syndrome

TAVI is associated with extreme shear stress across the stenotic native valve prior to implantation and rapidly changing flow conditions following valve deployment. High shear stress promotes vWF unfolding and excessive ADAMTS13-mediated cleavage of high-molecular-weight multimers, frequently resulting in acquired (AVWS). This molecular phenomenon has been linked to peri-procedural and postoperative bleeding complications, particularly gastrointestinal bleeding. In this setting, reduced platelet-dependent thrombus formation is captured by decreased PL chip parameters in T-TAS, reflecting impaired vWF-mediated platelet adhesion under high shear conditions [30].

#### 4.3.5. EVAR and TEVAR: Low Shear Stress, Flow Stagnation, and Fibrin-Dominant Thrombosis

In contrast, EVAR and TEVAR primarily alter flow dynamics by excluding aneurysmal segments and creating regions of low shear stress and flow stagnation, particularly within residual aneurysm sacs or false lumens. These conditions favor fibrin-rich thrombus formation with a relatively reduced dependence on vWF-mediated platelet adhesion. Endothelial injury induced by stent-graft deployment further enhances coagulation cascade activation. Within this context, the AR chip of T-TAS, which integrates platelet function with tissue-factor-driven coagulation under lower shear stress, is more sensitive to detecting increased thrombogenic potential. Elevated AR chip readouts may therefore reflect a prothrombotic state following EVAR or TEVAR, even in the absence of abnormalities in conventional coagulation tests [31].

#### 4.3.6. Clinical Integration of T-TAS in Endovascular Aortic Procedures

**Figure 5:**
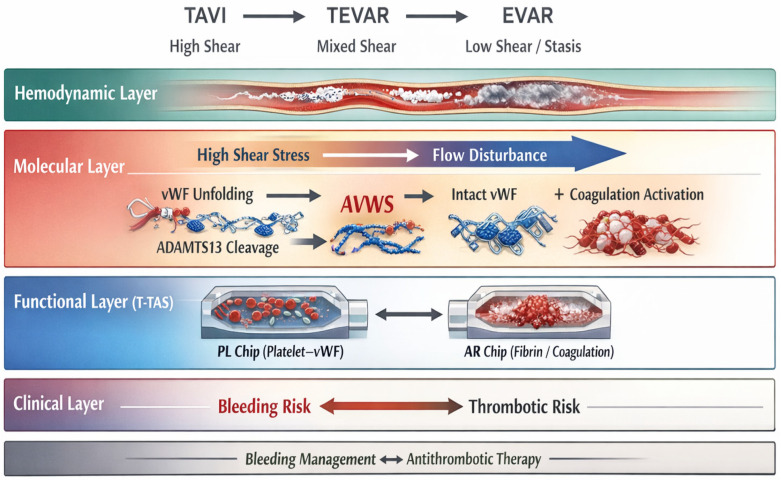
Molecular—clinical continuum Tavi–Tevar–Evar.

By differentially capturing shear-dependent platelet dysfunction (PL chip) and fibrin-dominant coagulation activation (AR chip), T-TAS provides functional platform to assess the molecular consequences of vWF–ADAMTS13 imbalance across distinct aortic interventions. This integrative approach may support individualized risk stratification, guide peri-procedural antithrombotic management, and improve monitoring of hemostatic recovery following TAVI, EVAR, and TEVAR [32] (Table 3 and Figure 5).

This comparison highlights that TAVI predominantly affects the platelet–vWF axis through extreme shear stress and ADAMTS13-mediated vWF degradation, making the PL chip particularly informative. In contrast, EVAR and TEVAR generate low-shear or disturbed flow environments that favor fibrin-dominant thrombosis, which is more accurately captured by AR chip readouts. T-TAS therefore enables procedure-specific functional assessment of hemostatic imbalance not reflected by conventional coagulation assays (Figure 5).

#### 4.3.7. Shear Stress–Dependent Hemostasis and the vWF–ADAMTS13 Axis in Aortopathies: Implications for T-TAS

Hemostasis in the aorta is strongly dependent on local hemodynamic conditions, particularly shear stress, which has a decisive influence on platelet activation, von Willebrand factor (vWF) conformation, and coagulation pathways. High shear stress, characteristic of stenotic or turbulent aortic flow, induces elongation and unfolding of vWF multimers, exposing the A2 domain and enabling proteolytic cleavage by ADAMTS13, thereby regulating thrombotic potential [6,7,8,9,10,11,12,13,14,15,16,17,18,19,20,38,58,59,60,61,62,63,64,65,66]. This shear-dependent mechanism is essential for maintaining hemostatic balance and preventing pathological microvascular thrombosis. In contrast, low shear stress and flow stagnation, as observed in dilated aortas or aneurysmal segments, favor fibrin-dominant clot formation driven by coagulation cascade activation rather than platelet–vWF interactions. These distinct hemodynamic environments are reflected in the functional readouts of the (T-TAS), a microfluidic assay that evaluates thrombus formation under controlled flow conditions using whole blood [33].

Aortopathies and aortic interventions represent clinical scenarios characterized by profound alterations in shear stress and vWF biology. In patients with severe aortic stenosis undergoing transcatheter (TAVI), excessive shear stress across the stenotic valve leads to enhanced ADAMTS13-mediated cleavage of vWF and the development of AVWS, which has been shown to be partially reversed following valve replacement. Functional assessment using T-TAS has demonstrated changes in thrombogenic activity and platelet function before and after TAVI, supporting its potential role in peri-procedural hemostatic evaluation. Similarly, thoracic and abdominal endovascular aortic repair (TEVAR and EVAR) modify local flow patterns, potentially shifting the hemostatic balance from high-shear platelet-driven mechanisms toward low-shear, coagulation-dominant thrombosis. These alterations may contribute to both bleeding and thrombotic complications observed after endovascular interventions. In this context, T-TAS offers a platform to assess the global hemostatic phenotype under flow, integrating the molecular consequences of shear stress on the vWF–ADAMTS13 axis with clinically relevant thrombus formation dynamics. Collectively, these observations support the concept of a molecular-to-clinical continuum linking shear stress, vWF biology, and thrombus formation in aortopathies. T-TAS emerges as a promising tool for risk stratification, peri-operative assessment, and individualized antithrombotic management in patients undergoing aortic interventions [9,34,35,36,37,38,39,40,41,42,43].

#### 4.3.8. FBN1 and Marfan Syndrome

**Figure 6:**
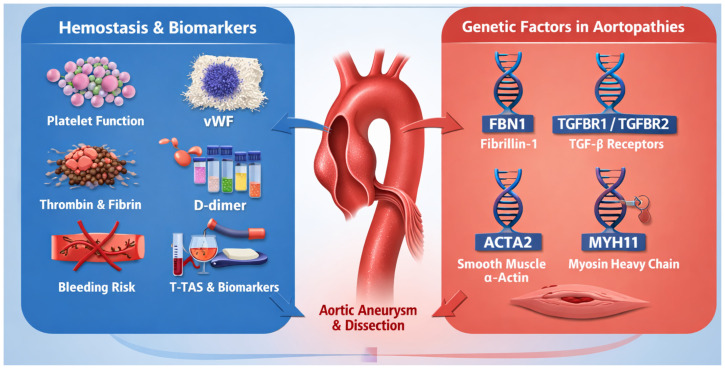
Conceptual relationship between genetic determinants of aortopathy and systemic hemostatic phenotype. Schematic overview of the interplay between genetic factors underlying aortopathies and circulating hemostatic processes. On the right, key genes associated with heritable thoracic aortic disease are shown, including *FBN1* (fibrillin-1), *TGFBR1/TGFBR2* (transforming growth factor-β receptors), *ACTA2* (smooth muscle α-actin), and *MYH11* (smooth muscle myosin heavy chain). These genes primarily affect extracellular matrix integrity, smooth muscle cell function, and TGF-β signaling pathways, leading to structural weakening of the aortic wall and susceptibility to aneurysm formation and dissection. On the left, systemic components of hemostasis are illustrated, including platelet function, von Willebrand factor (vWF), thrombin–fibrin generation, and circulating biomarkers such as D-dimer. Functional assays, including flow-based systems (e.g., T-TAS), reflect the integrated thrombotic or bleeding phenotype of circulating blood under dynamic conditions. Importantly, no direct molecular link is implied between these genetic mutations and systemic hemostatic alterations. Instead, the relationship is conceptual and likely indirect, mediated through chronic changes in vascular structure and hemodynamics. Genetic defects lead to alterations in aortic geometry, compliance, and flow patterns, which may secondarily influence endothelial behavior, shear-stress-dependent platelet activation, and coagulation processes. Thus, any association between aortopathy-related genes and systemic hemostatic profiles should be interpreted as potential rather than causative, requiring validation in prospective and mechanistic studies. This figure emphasizes a multimodal framework in which genetic, structural, and functional data are integrated to better understand disease pathophysiology.

The *FBN1* gene, located on chromosome 15q21.1, encodes fibrillin-1, a large extracellular matrix glycoprotein essential for the formation of calcium-binding microfibrils that provide structural support and elasticity to connective tissues, including the aortic wall. These microfibrils are crucial for the mechanical integrity of elastic vessels and for regulating transforming growth factor β (TGF-β) signaling by sequestering latent TGF-β complexes in the extracellular matrix. Pathogenic FBN1 variants are the primary cause of Marfan syndrome (MFS), an autosomal dominant connective tissue disorder characterized by a high predisposition to thoracic aortic aneurysms and dissections, as well as systemic features such as skeletal and ocular abnormalities [46,47,48,49,50,51,52,53,54,55,56,57,58]. In MFS, defective fibrillin-1 leads to abnormal microfibril structure and dysregulated TGF-β activation, contributing to progressive aortic root dilation and loss of elastic fiber integrity. Extracellular matrix dysfunction may also indirectly affect local hemodynamics and platelet activation, thereby altering hemostatic balance. FBN1 variants are frequently identified in patients with (HTAD); however, their penetrance for aortic events varies across populations and may be influenced by modifier genes and environmental factors [47,48,49,50,51,52,53,54,55,56,57,58] (Figure 6).

#### 4.3.9. TGFBR1/TGFBR2 and Loeys–Dietz Syndrome

Mutations in *TGFBR1* and *TGFBR2*, which encode Type I and Type II TGF-β receptors, cause Loeys–Dietz syndrome (LDS), a heritable connective tissue disorder characterized by aggressive, early-onset thoracic aortic aneurysms and dissections, arterial tortuosity, and additional skeletal and craniofacial abnormalities. Mutations in genes such as *FBN1*, *TGFBR1*, *TGFBR2*, *ACTA2*, and *MYH11* are well-established causes of heritable aortopathies. These genetic alterations primarily affect vascular wall structure and signaling pathways. Potential downstream effects on systemic hemostasis remain indirect and incompletely understood. In the vascular wall, TGF-β signaling maintains vascular smooth muscle cell (VSMC) homeostasis and extracellular matrix composition. Pathogenic heterozygous variants in *TGFBR1* or *TGFBR2* directly disrupt receptor function, yet paradoxically often lead to upregulation of TGF-β signaling within the aortic wall, contributing to vascular remodeling and disease progression. Dysregulated signaling may also influence the balance of pro- and anti-thrombotic signals in the vessel lumen, modulating platelet-vessel wall interactions. Genetic studies indicate that *TGFBR1/2* variants not only underlie LDS but can also modify phenotypic expression in syndromes with overlapping features, highlighting the complex role of TGF-β signaling in aortopathy [48,49] (Figure 6).

#### 4.3.10. ACTA2

Located on chromosome 10q23.31, encodes smooth muscle α-actin (αSMA), a major cytoskeletal protein of (VSMCs) that is essential for contractility, vessel tone, and mechanical responsiveness to hemodynamic stress. Pathogenic *ACTA2* variants impair actin filament assembly and VSMC contraction, leading to defective vascular wall remodeling, altered mechanotransduction, and modified local flow conditions, which can influence platelet activation and overall hemostasis. Mutations in *ACTA2* are a common cause of (FTAAD), representing a significant proportion of non-syndromic (HTAD). Loss of smooth muscle contractile integrity predisposes to aneurysm formation, aortic dissection, and other vascular pathologies, including early-onset cerebrovascular disease. Functional studies also suggest that *ACTA2* mutations may enhance TGF-β signaling in VSMCs, indicating that defects in the contractile apparatus can have secondary effects on extracellular matrix dynamics and contribute to aortic disease progression [51,52,53,54,55,56,57,58] (Figure 6).

#### 4.3.11. MYH11—Smooth Muscle Myosin Heavy Chain

The *MYH11* gene, located on chromosome 16p13, encodes the *smooth muscle myosin heavy chain 11*, a core component of the contractile apparatus of VSMCs. MYH11 interacts with α-actin to generate the mechanical force necessary for smooth muscle contraction. Pathogenic MYH11 variants are implicated in a subset of non-syndromic FTAAD cases, often associated with persistent ductus arteriosus and other vascular anomalies. Similar to ACTA2, MYH11 mutations result in compromised contractile function and remodeling of the vessel wall, which, in turn, can modify local hemodynamics and contribute to aneurysm susceptibility. Some studies have demonstrated that defective myosin assembly may influence TGF-β signaling pathways, further linking smooth muscle contractile gene dysfunction to broader molecular mechanisms of aortic disease [54,55,56,57,58] (Figure 6).

#### 4.3.12. Clinical and Pathophysiological Implications

Collectively, these genes highlight two major molecular pathways in aortopathy: disruption of the extracellular matrix (FBN1) and dysfunction of smooth muscle cell contractility and TGF-β signaling (*TGFBR1/2*, *ACTA2*, *MYH11*). Variants in these genes not only determine structural vulnerability of the aortic wall but also influence dynamic cellular responses to mechanical stress and signaling cues that are central to aneurysm formation, progression, and dissection [47,48,49,50,51,52,53,54,55,56,57,58].

Genotype–phenotype correlation studies in large cohorts of patients with HTAD have revealed that TGF-β pathway-related gene variants are often associated with more aggressive disease progression and earlier vascular events compared with FBN1 variants, which may show varied penetrance and reflect distinct pathobiological effects. Furthermore, polymorphic variants in these genes have been linked to aortic dissection susceptibility in population studies, indicating that not only rare high-impact mutations but also common genetic variants may modulate individual risk profiles [47,48,49,50,51,52,53,54,55,56,57,58].

#### 4.3.13. Integration with Hemostasis Assessment

Understanding the molecular basis of aortic wall vulnerability provides context for applying hemostasis assays such as the Total Thrombus-Formation Analysis System (T-TAS) in aortopathy patients. Genetic defects affecting aortic structure and signaling pathways may influence local mechanical forces and endothelial–platelet interactions, potentially altering thrombotic and bleeding risks in these patients. Integration of genetic profiling with dynamic hemostasis assays could inform personalized risk stratification and therapeutic planning, especially in the perioperative setting or in individuals receiving antithrombotic therapy [57,58,59,60,61,62,63].

#### 4.3.14. Potential Research Directions

Key avenues for research include:

1. Correlation of T-TAS parameters with genetic mutations—linking hemostatic profiles to specific variants (*FBN1*, *TGFBR1/2*, *ACTA2*, *MYH11*) to identify high-risk thrombotic or bleeding phenotypes [40,41,42,43,44,45,46,47,48,49,50,51,52,53,54,55,56,57,58,59,60,61,62,63].
2. Identification of high-risk hemostatic phenotypes—integrating T-TAS data with genetic profiling to pinpoint patients at elevated risk for hemorrhagic or thrombotic complications [43,44,45,46,47,48,49,50,51,52,53,54,55,56,57,58,59,60,61,62,63].
3. Development of personalized hemostatic and surgical strategies—understanding individual hemostatic variability may inform tailored dosing of anticoagulants or antiplatelet agents, as well as optimized surgical planning for genetically mediated aortopathies [46,47,48,49,50,51,52,53,54,55,56,57,58,59,60,61,62,63].

#### 4.3.15. Can T-TAS Improve Risk Stratification or Management in Aortopathies?

However, the application of T-TAS in the context of aortopathies remains entirely exploratory. To date, no clinical trials or large cohort studies have directly assessed T-TAS metrics in patients with thoracic aortic aneurysms, dissections, or genetic aortopathies. While mechanistic rationale exists—high shear stress in diseased aortas can induce vWF unfolding, platelet activation, and altered thrombus formation—the translation of these observations into actionable clinical guidance has not been demonstrated [9,10,11]. Practical limitations, including assay standardization, feasibility in urgent scenarios (e.g., acute dissection), and interpretation of peripheral whole-blood measurements relative to local aortic hemodynamics, further restrict immediate clinical application.

Given these constraints, T-TAS cannot currently be considered a validated tool for risk stratification or management in aortopathies. Instead, its potential lies in hypothesis generation and mechanistic investigation, where it may be used to explore how genetic variants, structural abnormalities, and hemodynamic perturbations collectively influence systemic hemostasis. Future studies and imaging data may determine whether functional hemostatic profiling can add predictive value beyond conventional assessments, particularly in perioperative or high-risk patients.

T-TAS represents a promising research tool for exploring systemic hemostatic consequences of aortic disease, but its role in personalized risk stratification or clinical management remains speculative. Any potential translation into practice will require rigorous prospective studies, standardized protocols, and careful correlation with structural and genetic markers of aortopathy [6,7,8,9,10,11,12,13,14,15,16,17,18,19,20,38,39,40,41,42,43,44,45,46,47,48,49,50,51,52,53,54,55,56,57,58,59,60,61,62,63,64,65,66,67,68,69,70,71,72]. It must be emphasized that peripheral whole-blood measurements may not accurately reflect focal aortic hemodynamics. Extrapolation from systemic assays to localized structural vascular pathology requires caution. Moreover, the feasibility of implementing flow-dependent assays such as T-TAS in acute aortic syndromes remains uncertain due to time constraints and lack of standardized perioperative protocols. Accordingly, any proposed role of T-TAS in aortopathies remains investigational and should not be interpreted as clinically validated at this stage.

Peripheral whole-blood measurements may not accurately reflect focal aortic hemodynamics, which are spatially heterogeneous and influenced by local vascular geometry [10,11,12,20]. Extrapolation from systemic assays to localized structural vascular pathology therefore requires caution. Furthermore, although T-TAS has demonstrated clinical relevance in coronary artery disease, atrial fibrillation, and device-related populations [6,7,8,9,10,11,12,13,14,15,16,17,18,19,20,21,22], its role in aortopathies remains unvalidated [8,9]. Accordingly, any proposed clinical application should be considered investigational pending prospective outcome-based studies.

Particular attention should also be paid to two-way fluid–structure interaction (FSI). The analysis of pulse wave propagation in the aorta using FSI models represents a significant advancement over conventional approaches based solely on computational fluid dynamics (CFD). Unlike standard CFD models, which typically assume rigid vessel walls, FSI incorporates the bidirectional coupling between blood flow and arterial wall deformation. This enables a more physiologically accurate representation of key phenomena, including pulse wave propagation, wave reflection, and spatial variations in aortic compliance [86,87,88]. Consequently, FSI models provide improved estimation of hemodynamic parameters, particularly wall shear stress (WSS), which is strongly influenced by dynamic changes in vessel geometry.

In the context of aortopathies, where structural alterations of the vessel wall—such as aneurysmal dilation or aortic dissection—play a central role, FSI modeling allows for a more comprehensive assessment of the interplay between hemodynamics and wall mechanics. Traditional CFD models may overestimate WSS and fail to capture the damping effect of the compliant aortic wall on pulse wave transmission, thereby limiting their predictive value in clinical applications [89,90]. For this reason, FSI is increasingly regarded as a more appropriate framework for investigating disease progression in the aorta.

A major challenge in FSI modeling, however, lies in the accurate characterization of the mechanical properties of the aortic wall. Aortic tissue exhibits complex behavior, including nonlinearity, anisotropy, and heterogeneity due to its multilayered structure (intima, media, and adventitia) [91,92]. Furthermore, pathological conditions introduce additional alterations, such as elastin degradation, collagen remodeling, and calcification, all of which significantly affect local stiffness and mechanical response [93]. The lack of reliable in vivo measurements necessitates the use of simplified constitutive models, which introduces uncertainty into simulation outcomes.

Another critical aspect is the implementation of patient-specific models, which require individualized input data to accurately reproduce physiological hemodynamic conditions. These include inlet flow waveforms (e.g., derived from 4D flow MRI), arterial pressure measurements, and high-resolution aortic geometry obtained from imaging modalities such as computed tomography (CT) or magnetic resonance imaging (MRI) [94,95]. Without such personalization, computational models lose their quantitative reliability and remain primarily qualitative. In particular, the appropriate definition of boundary conditions plays a crucial role in determining flow distribution, pulse wave characteristics, and local hemodynamic indices.

Hemodynamic metrics such as time-averaged wall shear stress (TAWSS) and related shear-based indices (e.g., time-averaged shear (TAS)) are of particular importance in assessing thrombotic risk in aortopathies. Regions characterized by low TAWSS are associated with prolonged blood residence time, promoting platelet activation, endothelial dysfunction, and thrombus formation [96,97]. By accounting for vessel wall motion, FSI models enable more accurate identification of flow recirculation and stagnation zones, which are critical in the pathophysiology of thrombogenesis. Compared with rigid-wall CFD simulations, FSI therefore provides more reliable indicators of thrombotic risk.

From a clinical perspective, the personalization of computational models through individualized boundary conditions and vessel wall properties offers substantial potential for supporting medical decision-making. Such models may assist in surgical planning (e.g., determining the extent of aortic repair or optimizing stent-graft deployment), as well as in evaluating the need for anticoagulant therapy [98,99]. Moreover, FSI-based simulations can be used to predict disease progression and to assess the hemodynamic impact of different therapeutic strategies.

Two-way FSI modeling constitutes an advanced analytical framework that significantly enhances the physiological realism of hemodynamic simulations compared to traditional CFD approaches. Despite existing limitations—primarily related to uncertainties in material properties and the availability of patient-specific data—FSI holds considerable promise for improving diagnostic accuracy and risk stratification in patients with aortopathies.

## 5. Future Directions and Conclusions

### 5.1. Future Directions

Despite these limitations, T-TAS provides a promising platform for translational investigation, particularly when combined with multimodal assessments including genetic profiling, imaging, and potentially other hemostatic assays. Prospective studies should aim to:

1. Correlate T-TAS metrics with aortic diameter, flow patterns, and genotype in patients with heritable or sporadic aortopathies.
2. Compare T-TAS with standard viscoelastic assays (TEG, ROTEM) to identify flow-dependent signals.
3. Assess the predictive value of T-TAS for perioperative bleeding, thrombosis, or aortic complications in controlled cohorts.

By situating T-TAS within a hypothesis-driven, multimodal research framework, future studies may determine whether functional hemostatic assessment can complement traditional imaging and genetic risk stratification in aortic disease. Future research should focus on prospective studies evaluating T-TAS in patients with aortic aneurysm and dissection, correlation with advanced imaging-based flow analysis, and development of endothelialized microfluidic chips. Such advances may establish T-TAS as a valuable tool for personalized hemostatic assessment in aortic disease. Future research should investigate how T-TAS parameters (e.g., AUC values from PL and AR chips) correlate with:

- Presence or extent of aortic mural thrombi;
- Clinical outcomes such as embolic events;
- Response to antithrombotic interventions in aortic disease settings.

AUC quantifies platelet thrombus formation, representing the total thrombogenicity by measuring the area under the flow pressure–time curve, reflecting the speed and stability of clot formation, with lower AUC indicating impaired platelet function (primary hemostasis) and higher AUC suggesting normal or enhanced function.

#### 5.1.1. Aortopathies in Athletes: Implications for Physical Performance, Hemostatic Function, and Spatially Informed Health Management

In the context of sports science and physical performance evaluation, understanding the interaction between athletic activity and aortic pathology is crucial for safe participation, personalized risk stratification, and population-level health management.

Elite and lifelong athletes undergo substantial cardiovascular remodeling in response to chronic hemodynamic load. While the myocardium adapts robustly to repeated exercise stimuli, evidence suggests that the aorta may also exhibit adaptive—or in some cases maladaptive—changes. A systematic meta-analysis demonstrates that elite athletes may have slightly larger absolute aortic root dimensions compared with non-athletic controls; however, when indexed to body surface area (BSA), these differences are not consistently significant across disciplines and sexes, highlighting the need for proper normalization in clinical assessment [6,7,8,9,10,11,12,13,14,15,16,17,18,19,20,21,22,27,66,74,75,76,77,78,79,80].

#### 5.1.2. Aortic Size and Athletic Activity

Although relative aortic enlargement appears uncommon among young competitive athletes, studies in older cohorts and masters athletes show more variation. For example, among competitive rowers and runners aged 50–75 years, up to 21–31% exhibited aortic diameters ≥ 40 mm, suggesting that long-term endurance exercise may influence aortic dimensions over decades [6,7,8,9,10,11,12,13,14,15,16,17,18,19,20,21,22,27,66,67,68,69,70,71,72,73,74,75,76,77,78,79,80]. Similar findings in former elite contact sport athletes further emphasize that exercise type, intensity, and cumulative exposure may affect the aortic wall’s structure over time.

In congenital or genetic aortopathies, sport participation guidelines are inherently conservative. Scientific societies recommend shared decision-making (SDM) when considering competitive sports in individuals with (HTAD) or significant dilation, with participation thresholds influenced by aortic size, genetic diagnosis, and exercise hemodynamics [6,7,8,9,10,11,12,13,14,15,16,17,18,19,20,21,22,27,66,67,68,69,70,71,72,73,74,75,76,77,78,79,80]. Although competitive endurance or strength sports are generally considered inappropriate for those with large aneurysms or post-dissection status, active low-to-moderate intensity activities may be acceptable in select individuals with HTAD and normal aortic dimensions after careful SDM [6,7,8,9,10,11,12,13,14,15,16,17,18,19,20,21,22,27,66,67,68,69,70,71,72,73,74,75,76,77,78,79,80].

#### 5.1.3. Hemostatic Considerations in Athletes and Aortopathy

Exercise induces acute changes in coagulation and fibrinolysis. Prolonged or intense physical activity can lead to hemoconcentration, increased blood viscosity, and transient prothrombotic states, although regular exercise is generally associated with reduced baseline cardiovascular risk [6,7,8,9,10,11,12,13,14,15,16,17,18,19,20,21,22,27,66,67,68,69,70,71,72,73,74,75,76,77,78,79,80,81,82,83].

Within athletes, the subtle balance between pro- and anticoagulant forces can be influenced by dehydration, inflammation, and physiologic stress, making hemostatic profiling relevant not only to pathology but also to performance and recovery research.

In the specific setting of aortopathy, shear stress—a determinant of endothelial function and vWF biology—is particularly relevant. High wall shear stress in dilated or structurally compromised aortas may contribute to vWF multimer unfolding and altered thrombotic potential, which is central to aortic wall pathology and thrombo-hemorrhagic risk from a mechanistic standpoint. However, direct clinical evidence of exercise-induced systemic hemostatic disruption in aortopathy is lacking, and current guidelines do not incorporate routine dynamic functional assays.

#### 5.1.4. Functional Hemostasis Testing and T-TAS in Athletic Populations

The Total Thrombus-Formation Analysis System (T-TAS) is a microfluidic, flow-dependent assay capable of quantifying thrombus formation in whole blood under simulated arterial shear conditions. It has been applied in cardiovascular research to assess thrombogenicity and bleeding risk in various settings, including coronary artery disease and procedural cohorts [7,8,9]. Its offers feature—evaluation under defined flow—distinguishes it from static tests like PT, aPTT, or viscoelastic assays (TEG/ROTEM), potentially capturing flow-dependent platelet-coagulation dynamics influenced by exercise and vascular adaptation [6,7,8,9,10,11,12,13,14,15,16,17,18,19,20,21,22,27,66,67,68,69,70,71,72,73,74].

In athletes, T-TAS could theoretically serve as a research tool to investigate:

1. Functional hemostatic profiles in individuals with or without aortopathy across training loads and sport types, elucidating whether dynamic thrombus formation under flow differs between athlete subgroups or with genetic predisposition.
2. The interaction between structural aortic changes (e.g., mild dilation) and systemic hemostatic behavior under physiologic shear, which may help refine risk models beyond static measures.
3. Post-exertion or recovery hemostatic responses, potentially offering insights into optimal training loads and individualized conditioning.

However, it must be emphasized that no published studies to date have specifically used T-TAS to assess athletes, nor have they linked T-TAS profiles to aortic dimensions, exercise history, or clinical outcomes in this population. As such, the application of T-TAS remains speculative and primarily a tool for hypothesis generation rather than clinical practice at this stage [6,7,8,9,10,11,12,13,14,15,16,17,18,19,20,21,22,27,66,67,68,69,70,71,72,73,74].

#### 5.1.5. Geographical and Population Health Dimensions

From the perspective of Spatial Management and Socio-Economic Geography, aortic disease in athletes intersects with population health, healthcare resource allocation, and regional policy on sports participation. The prevalence of aortic dilation and structural variants among athletes may vary by region due to genetic, environmental, and socioeconomic determinants. For example, access to advanced cardiac imaging and long-term athlete monitoring programs differs substantially across countries and even within urban vs. rural settings, affecting the detection and management of aortopathy.

Large-scale screening initiatives and registry data can inform geographically stratified risk models that account for regional variations in athletic participation rates, demographic factors, and healthcare infrastructure, enabling better targeted surveillance strategies for athletes at risk of aortic complications. Moreover, mapping the distribution of genetic variants associated with HTAD (e.g., in *FBN1*, *TGFBR1/2*, *ACTA2*) could inform community-specific prevention and education programs aimed at safe physical activity engagement based on local genetic epidemiology [6,7,8,9,10,11,12,13,14,15,16,17,18,19,20,21,22,66,67,68,69,70,71,72,73,74,82].

#### 5.1.6. Practical Clinical and Research Implications

For physical culture sciences, the dynamic between training adaptation and aortic remodeling underscores the necessity of individualized athlete assessment, incorporating structural imaging (echocardiography, MRI), genetic evaluation when indicated, and functional hemostatic research. Shared decision-making frameworks, guided by contemporary guidelines and nuanced by athlete goals and risk tolerance, remain central to clinical practice [6,7,8,9,10,11,12,13,14,15,16,17,18,19,20,21,22,66,67,68,69,70,71,72,73,74,82].

Future research agendas should include:

- Longitudinal cohort studies to track aortic dimensions and functional hemostatic changes in athletes over time.
- Integration of flow-dependent assays like T-TAS with imaging and genetic data to explore mechanistic links and develop predictive models for adverse events.
- Geographically diverse sampling to understand regional variation in aortic adaptation and associated risks among athletic populations.

### 5.2. Conclusions

Aortopathies are primarily structural disorders of the aortic wall; however, the associated alterations in vascular geometry and compliance lead to significant disturbances in blood flow, including abnormal shear stress and turbulence. These hemodynamic changes may influence platelet activation, coagulation pathways, and the overall systemic hemostatic phenotype, suggesting a potential link between aortic pathology and flow-dependent thrombus formation.

The Total Thrombus-Formation Analysis System (T-TAS) provides a dynamic, flow-based assessment of hemostasis that captures the integrated behavior of circulating blood components under physiologically relevant conditions. In the context of aortopathies, its application remains exploratory and should be interpreted as a functional, potential approach rather than a diagnostic or risk-stratification tool.

Whether these functional alterations are consistently present and clinically meaningful in patients with aortic disease remains uncertain.

Future research should focus on prospective, well-characterized studies integrating imaging-based assessment of aortic structure and hemodynamics, genetic profiling, and flow-dependent functional hemostatic testing. Such multimodal approaches may help clarify whether systemic thrombus formation patterns can serve as meaningful markers of disease phenotype, progression, or clinical risk in aortopathies.

## 6. Methods: Literature Search and Selection Criteria

### 6.1. Study Design

This study is a narrative review aimed at providing a comprehensive and integrative overview of the potential application of the Total Thrombus-Formation Analysis System (T-TAS) in the evaluation of hemostatic disturbances in patients with aortopathies. Particular emphasis was placed on genetically mediated conditions, including Marfan syndrome, Loeys–Dietz syndrome, and aortic diseases associated with mutations in *FBN1*, *TGFBR1/2*, *ACTA2*, and *MYH11*.

The objective was to synthesize mechanistic, translational, and clinical evidence, highlighting both the potential utility and limitations of T-TAS within the context of aortic disease, while identifying current knowledge gaps and future research directions.

### 6.2. Search Strategy

A structured literature search was conducted using the electronic databases PubMed, Scopus, and Web of Science. The search covered publications from January 2000 to January 2025.

The search strategy incorporated combinations of keywords and Medical Subject Headings (MeSH), including:

- “T-TAS” OR “Total Thrombus-Formation Analysis System”;
- “hemostasis” OR “platelet function” OR “coagulation”;
- “aortopathy” OR “thoracic aortic aneurysm” OR “aortic dissection”;
- “Marfan syndrome” OR “Loeys–Dietz syndrome” OR “FBN1” OR “TGFBR1” OR “TGFBR2” OR “ACTA2” OR “MYH11”.

Boolean operators (AND, OR) were used to refine the search strategy (e.g., “T-TAS AND aortopathy” OR “T-TAS AND thoracic aortic aneurysm”).

Additionally, reference lists of relevant articles were manually screened to identify studies not captured in the primary database search.

### 6.3. Eligibility Criteria

Inclusion Criteria

Studies were included if they met the following criteria:

1. Original research articles, clinical studies, or reviews addressing T-TAS or other flow-dependent hemostatic assays in cardiovascular disease.
2. Studies providing mechanistic, translational, or functional insights into platelet activation, coagulation pathways, or von Willebrand factor (vWF) dynamics relevant to aortic pathology.
3. Publications available in English with full-text access.

Exclusion Criteria

Studies were excluded if they met any of the following:

1. Studies not involving human subjects or lacking translational cardiovascular relevance (e.g., purely in vitro studies unrelated to T-TAS or aortic disease).
2. Case reports without meaningful mechanistic or functional hemostatic data.
3. Articles without full-text availability or published in languages other than English.

### 6.4. Study Selection

After removal of duplicates, titles and abstracts were screened independently by two reviewers to assess relevance. Studies deemed potentially eligible underwent full-text review.

Discrepancies between reviewers were resolved through discussion and consensus. Studies were selected based on their relevance to:

- Aortopathy and associated hemodynamic disturbances;
- Functional hemostasis assessment;
- The clinical or experimental application of T-TAS.

### 6.5. Data Extraction and Management

Relevant data were extracted manually from selected studies and organized into thematic categories, including:

- Study design and population characteristics;
- Type of hemostatic assessment (e.g., T-TAS, platelet function tests, coagulation assays);
- Key findings related to thrombotic or bleeding risk;
- Mechanistic insights into flow-dependent hemostasis;
- Relevance to aortic pathology or vascular remodeling.

Given the narrative nature of the review, no standardized data extraction forms or software tools were applied.

### 6.6. Data Synthesis

A qualitative synthesis approach was employed. The selected literature was analyzed and integrated into a conceptual framework linking:

- Structural abnormalities of the aorta;
- Genetic determinants of aortopathy;
- Hemodynamic disturbances (e.g., shear stress, turbulence);
- Systemic hemostatic alterations.

The review emphasizes potential interpretations, particularly regarding the potential role of T-TAS in capturing flow-dependent thrombotic phenotypes in aortic disease.

### 6.7. Quality Assessment

As this is a narrative review, a formal systematic quality assessment or meta-analysis was not performed.

Instead, studies were critically appraised based on:

- Methodological rigor;
- Relevance to cardiovascular and aortic pathology;
- Contribution to understanding flow-dependent hemostasis.

Preference was given to studies providing mechanistic insight, translational relevance, or clinical applicability.

### 6.8. Figure Design

Figures included in this review were designed as schematic and conceptual illustrations to support the interpretation of complex relationships between aortic structure, hemodynamics, genetic factors, and systemic hemostasis.

They do not represent direct experimental data but rather aim to:

- Summarize current knowledge;
- Illustrate proposed mechanisms;
- Highlight potential links between aortopathy and functional hemostatic assessment.

All graphical elements were created to enhance clarity and should be interpreted within the conceptual framework of the review.

All figures were created using BioRender (Scientific Publishing Software 2026 V1.0) and Adobe Illustrator 2026 (version 30.2).

### 6.9. Limitations of the Methodological Approach

This review has several inherent limitations. As a narrative review, it does not provide a systematic or quantitative synthesis of the literature and may be subject to selection bias.

The available evidence regarding T-TAS in aortopathies is limited, with most studies conducted in other cardiovascular settings, such as coronary artery disease or transcatheter interventions. Therefore, conclusions regarding its role in aortic disease remain speculative and potential.

Additionally, heterogeneity in study design, patient populations, and hemostatic assays limits direct comparability between studies. The absence of large prospective trials further restricts the ability to draw definitive clinical conclusions.

**Figure 7:**
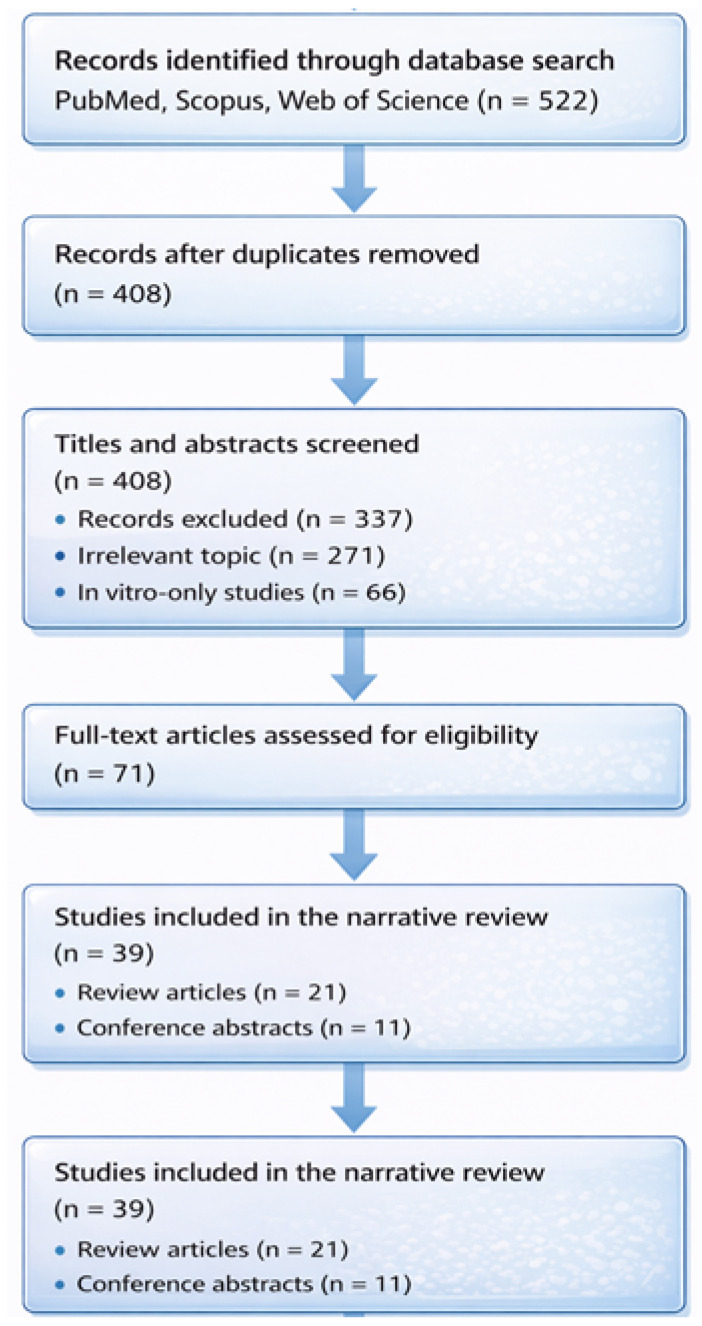
Flowchart of literature search and study selection process. Flow diagram illustrating the identification, screening, eligibility assessment, and inclusion of studies in this narrative review. A total of 522 records were identified through database searching (PubMed, Scopus, and Web of Science). After removal of duplicates (n = 408), titles and abstracts were screened, leading to the exclusion of 337 records due to irrelevance or lack of translational relevance. Full-text articles (n = 71) were assessed for eligibility, and 39 studies were ultimately included in the review. Included studies comprised original research articles, reviews, and conference abstracts that provided mechanistic, translational, or clinical insights into flow-dependent hemostasis and its potential relevance to aortopathies.

The literature selection process is summarized in Figure 7, which illustrates the identification, screening, eligibility assessment, and final inclusion of studies considered in this review.

## Figures and Tables

**Table 1:** Comparison of T-TAS and Conventional Hemostasis Tests.

Feature	T-TAS	Conventional Hemostasis Tests (PT, aPTT, Platelet Aggregometry)
Sample type	Whole blood [6,7,8,9,21,34,45]	Plasma or platelet-rich plasma [5]
Flow conditions	Yes; microfluidic flow with controlled shear stress [7,21,34]	No; static assay conditions [5]
Platelet–coagulation interaction	Integrated, simultaneous assessment under flow [6,7,8,9,21]	Assessed separately using distinct assays [5]
Simulation of arterial conditions	Partial simulation of arterial shear conditions [7,20,21,33]	No simulation of physiological flow [5]
Sensitivity to shear-dependent mechanisms (e.g., vWF-mediated platelet adhesion)	High, due to flow-dependent thrombus formation [19,20,21,23,24,33,37]	Low to absent [5,33]
Ability to reflect endothelial or hemodynamic influences	Indirect and functional, via shear-dependent thrombus formation [7,10,11,21]	Minimal [5]
Clinical relevance in vascular disease	Potential; demonstrated mainly in coronary and device-related cardiovascular conditions [6,13,14,17,22,59,60,61,62,63,64,65,66,67]	Established primarily for systemic coagulation disorders [5]
Applicability in aortopathies	Investigational; no direct clinical validation to date [4,10,11,22]	Limited to exclusion of systemic coagulation abnormalities [1,5]
Turnaround time	Short (approximately 30–60 min) [6,7,45]	Short to moderate [5]
Current role in clinical practice	Adjunctive or research-oriented [8,9,45]	Standard diagnostic tools [5]

T-TAS—provides complementary functional information under flow conditions. Its clinical relevance in aortic diseases remains hypothetical and requires validation in dedicated clinical studies.

**Table 2:** Comparative table that highlights T-TAS alongside TEG, platelet aggregometry, and classical coagulation tests, emphasizing complementarity than superiority.

Assay	Measured Parameters	Flow Dependence	Distinctive/Complementary Value in Aortic Disease
T-TAS	Whole-blood thrombus formation integrating platelet adhesion, aggregation, and coagulation; occlusion time; thrombus growth	High shear/dynamic flow	Captures flow-dependent, integrative thrombus formation; sensitive to shear-mediated platelet dysfunction and the vWF–ADAMTS13 axis; reflects combined cellular and coagulation contributions relevant to disturbed aortic hemodynamics [6,7,8,9,19,20,21,23,24,25,26,27,28,29,30,31,32,33,34,35,36,37]
TEG/ROTEM	Clot initiation, propagation kinetics, clot strength, fibrinolysis	Low shear/near-static	Provides a global assessment of coagulation dynamics and clot mechanical properties; useful in perioperative, trauma, and interventional settings; complements T-TAS by characterizing clot stability rather than flow-dependent platelet adhesion [5,6,8]
Platelet Aggregometry	Platelet aggregation responses to specific agonists (e.g., ADP, collagen, arachidonic acid)	Static	Enables assessment of isolated platelet signaling pathways and agonist-specific defects; complements T-TAS by identifying pharmacological or receptor-level platelet dysfunction not captured under flow [5,8,9,13]
Classical Coagulation Tests (PT, aPTT, fibrinogen, factor assays)	Plasma clotting times and coagulation factor activity	None (static)	Standardized evaluation of intrinsic and extrinsic coagulation pathways; provides molecular baseline information essential for interpretation of global hemostatic assays; complements T-TAS by defining systemic coagulation status [1,5]

**Table 3:** Comparison of TAVI, EVAR, and TEVAR with Respect to Shear Stress, vWF–ADAMTS13 Axis, and T-TAS Readouts.

Feature	TAVI	EVAR	TEVAR
Primary hemodynamic alteration	Extreme supraphysiological shear stress across stenotic aortic valve before implantation, with rapid normalization after valve replacement [27,28,29,30,74]	Low shear stress and flow stagnation within the excluded aneurysm sac [10,11,43,73]	Low to heterogeneous shear stress, particularly within the false lumen or around the stent-graft [11,75,81,82]
Dominant shear profile	High arterial shear [29,33]	Low shear/flow stasis [10,11]	Mixed: low shear and disturbed flow [11,75]
vWF conformational changes	Shear-induced unfolding and proteolysis of high-molecular-weight vWF multimers [23,24,29,33,40]	Reduced shear-dependent unfolding of vWF multimers [10,43]	Limited or heterogeneous unfolding; persistence of larger multimers possible depending on flow remodeling [11,75]
ADAMTS13 activity	Accelerated cleavage of high-molecular-weight vWF multimers under high shear [23,24,37,38]	Relatively preserved ADAMTS13 activity [23,43]	Variable, dependent on post-procedural hemodynamic remodeling [11,75]
Typical vWF phenotype	Loss of high-molecular-weight multimers leading to acquired (AVWS) [25,26,27,28,29,30,74]	Relative preservation of vWF multimers [43,73]	Heterogeneous phenotype that may evolve over time [11,75]
Predominant hemostatic risk	Bleeding, particularly gastrointestinal bleeding (Heyde syndrome) [25,30,74]	Thrombosis, including mural or aneurysm sac thrombosis [3,4,11]	Thrombosis within the false lumen or around the stent graft [6,12,75,81]
Dominant thrombus composition	Platelet-dependent, vWF-mediated but functionally impaired thrombus formation [20,23,29]	Predominantly fibrin-rich thrombus [11,43]	Fibrin-dominant thrombus with variable platelet contribution [11,12,81]
Most informative T-TAS chip (hypothetical)	PL chip (platelet-dominant conditions) [14,22,65,66]	AR chip (combined platelet–coagulation assessment) [6,13]	AR chip > PL chip (exploratory) [6,13]
Expected PL chip findings	Reduced platelet thrombus formation reflecting AVWS and shear-induced platelet dysfunction [14,22,65]	Near-normal or mildly reduced platelet thrombus formation [6,13]	Variable, dependent on flow remodeling and platelet activation state [6,13]
Expected AR chip findings	Mild or secondary changes driven by coagulation rather than platelet defects [6,13]	Increased fibrin-rich thrombus formation reflecting prothrombotic milieu [6,13,43]	Increased thrombus formation in prothrombotic or inflammatory states [6,13]
Clinical utility of T-TAS	Detection of shear-dependent platelet dysfunction and AVWS; validated in TAVI and device-related cohorts [14,22,65,66]	Investigational identification of prothrombotic tendency following EVAR [6,13,43]	Investigational monitoring of thrombogenicity and hemostatic remodeling after TEVAR [6,13]

## Data Availability

No new data were created or analyzed in this study.

## Abbreviations

APTT	activated partial thromboplastin time
T-TAS	Total Thrombus-Formation Analysis System
PL	platelet chip
AR	atheroma chip
TAVI	aortic valve implantation
vWF	von Willebrand factor
GPIb	glycoprotein Ib
AVWS	von Willebrand syndrome
EVAR	endovascular
TEVAR	thoracic endovascular aortic repair
AUC	Area Under the Curve
PT	Prothrombin Time
OST	Occlusion Start Time
OT	Occlusion Time
AR (chip)	Atheroma chip (Atheroma chip (a microfluidic channel in T-TAS designed to assess primary and secondary hemostasis)
FTAAD	Familial Thoracic Aortic Aneurysm and Dissection
HTAD	Heritable Thoracic Aortic Disease
MFS	Marfan Syndrome
LDS	Loeys–Dietz Syndrome
FBN1	Fibrillin-1 gene
TGF-β	Transforming Growth Factor beta
TGFBR1	Transforming Growth Factor Beta Receptor 1
TGFBR2	Transforming Growth Factor Beta Receptor 2
ACTA2	Actin Alpha 2, Smooth Muscle
MYH11	Myosin Heavy Chain 11 (smooth muscle)
VSMC/VSMCs	Vascular Smooth Muscle Cell(s)
SMAD	SMAD family transcription factors
TEG	Thromboelastograp
ROTEM	Rotational Thromboelastometry
α-actin	(αSMA)
(FSI)	two-way fluid–structure interaction
(CFD)	computational fluid dynamics
(WSS)	wall shear stress
(CT)	computed tomography
(MRI)	magnetic resonance imaging
(TAWSS)	time-averaged wall shear stress
(TAS)	time-averaged shear

## References

[1] Erbel R., Aboyans V., Boileau C., Bossone E., Di Bartolomeo R., Eggebrecht H., Evangelista A., Falk V., Frank H., Gaemperli O. (2014). 2014 ESC Guidelines on the diagnosis and treatment of aortic diseases: Document covering acute and chronic aortic diseases of the thoracic and abdominal aorta of the adult. Eur. Heart J..

[2] Morris S.A., Flyer J.N., Yetman A.T., Quezada E., Cappella E.S., Dietz H.C., Milewicz D.M., Ouzounian M., Rigelsky C.M., Tierney S. (2024). Cardiovascular Management of Aortopathy in Children: A Scientific Statement from the American Heart Association. Circulation.

[3] Mendes D., Veiga C., Machado R., Sá-Pinto P., Almeida R. (2024). The Challenge of Managing a Primary Aortic Mural Thrombus: Outcomes and Technical Considerations. Int. J. Angiol..

[4] Komiya K., Imada S., Ujihara Y., Sugita S., Nakamura M. (2024). Predictive Methods for Thrombus Formation in the Treatment of Aortic Dissection and Cerebral Aneurysms: A Comprehensive Review. Bioengineering.

[5] Mendonça F.T., Matos S.H., Moreira L.G., Pereira I.L., Dino L.L., Braga M.B., Dias G.H. (2025). Accuracy of a Point-of-Care CoaguChek Test versus Standard Laboratory Coagulation Monitoring in Cardiac Surgery Involving Cardiopulmonary Bypass: Randomized Clinical Trial. Braz. J. Anesthesiol..

[6] Asiri A., Price J.M.J., Hazeldine J., McGee K.C., Sardeli A.V., Chen Y., Sullivan J., Moiemen N.S. (2024). Measurement of Platelet Thrombus Formation in Patients Following Severe Thermal Injury Using a Whole Blood Flow-Chip Based System. Platelets.

[7] Zheng K.L., Wallén H., Aradi D., Godschalk T.C., Hackeng C.M., Dahlen J.R., Ten Berg J.M. (2022). The Total Thrombus Formation (T-TAS) platelet (PL) assay, a novel test that evaluates whole blood platelet thrombus formation under physiological conditions. Platelets.

[8] Sikora J., Karczmarska-Wódzka A., Bugieda J., Sobczak P. (2021). The Use of Total Thrombus Formation Analysis System as a Tool to Assess Platelet Function in Bleeding and Thrombosis Risk—A Systematic Review. Int. J. Mol. Sci..

[9] Mansouritorghabeh H., Monard A., Heubel-Moenen F., Leentjens J., Stroobants A., Henskens Y. (2024). The Utility of Total Thrombus-Formation Analysis System (T-TAS) in the Thrombosis and Hemostasis Field: A Scoping Review. Int. J. Lab. Hematol..

[10] Guest A., Forneris A., Satriano A., Moore R.D., Di Martino E.S. (2025). Abdominal Aortic Aneurysm Classification Based on Dynamic Intraluminal Thrombus Analysis during Cardiac Cycle. J. Vasc. Surg. Cases Innov. Tech..

[11] Messou J.-C.E., Yeung K., Sudbrook E., Zhang J., Toursavadkohi S., Ucuzian A.A., Tubaldi E. (2024). Investigating the Role of Thrombosis and False Lumen Orbital Orientation in the Hemodynamics of Type B Aortic Dissection. Sci. Rep..

[12] DeRoo E., Stranz A., Yang H., Hsieh M., Se C., Zhou T. (2022). Endothelial dysfunction in the pathogenesis of abdominal aortic aneurysm. Biomolecules.

[13] Freitag K., Franke G.-N., Weise M., Herling C., Klöter T., Herling M., Siegemund A., Jentzsch M., Petros S., Henschler R. (2025). Total Thrombus Formation Analysis in Patients with Myeloid Neoplasia and Thrombocytopenia. Ann. Hematol..

[14] Lecchi A., La Marca S., Padovan L., Boscarino M., Peyvandi F., Tripodi A. (2024). Flow-Chamber Device (T-TAS) to Diagnose Patients Suspected of Platelet Function Defects. Blood Transfus..

[15] Tajima S., Kudo T., Mori D., Kitabayashi K. (2024). Floating Ascending Aortic Thrombus with Antiphospholipid Syndrome: A Case Report. Gen. Thorac. Cardiovasc. Surg. Cases.

[16] Mehta C., Raza F. (2024). In Situ Ascending Aortic Thrombus in a Patient with Metastatic Lung Adenocarcinoma and No Aortic Atherosclerosis or Cisplatin Exposure: A Case Report. J. Med. Case Rep..

[17] Mitsuse T., Kaikita K., Ishii M., Oimatsu Y., Nakanishi N., Ito M., Arima Y., Sueta D., Iwashita S., Fujisue K. (2020). Total Thrombus-Formation Analysis System can predict 1-year bleeding events in patients with coronary artery disease. J. Atheroscler. Thromb..

[18] Dimitrijevic Z.M., Apostolović S., Tasić D.D., Cvetković T.P., Lazarević M.V., Hozokawa K., Ågren A., Wallén H. (2026). Prediction of Bleeding and Thromboembolic Complications in Patients on Chronic Hemodialysis with the Total Thrombus-Formation Analysis System. Platelets.

[19] Nakajima Y., Yada K., Ogiwara K., Furukawa S., Shimonishi N., Shima M., Nogami K. (2021). A microchip flow-chamber assay screens congenital primary hemostasis disorders. Pediatr. Int..

[20] Okhota S., Kozlov S., Avtaeva Y., Melnikov I., Saburova O., Guria K., Matroze E., Gabbasov Z. (2023). Platelet Adhesion Mediated by von Willebrand Factor at High Shear Rates Is Associated with Premature Coronary Artery Disease. Biomedicines.

[21] Samanbar S., Piñeyroa J.A., Moreno-Castaño A.B., Pino M., Torramadé-Moix S., Martinez-Sanchez J., Lozano M., Sanz C., Escolar G., Díaz-Ricart M. (2023). T-TAS^®^01 as a new tool for the evaluation of hemostasis in thrombocytopenic patients after platelet transfusion. Blood Transfus..

[22] Varghese S.S., Hetu M., Bowman M., Herr J., Al-Turki M., Jaff Z., James P., Malik P., Payne D., Johri A.M. (2023). Johri Impact of transcatheter aortic valve implantation on circulating von Willebrand factor in patients with severe aortic stenosis. Haemophilia.

[23] Shaw R.J., Abrams S.T., Badu S., Toh C.-H., Dutt T. (2024). The Highs and Lows of ADAMTS13 Activity. J. Clin. Med..

[24] Makris M., Gavriilaki E., Ztriva E., Evangelidis P., Lefkou E., Vlachaki E., Bountola S., Perifanis V., Matsagkas M., Savopoulos C. (2025). Prospective Study of ADAMTS13 and von Willebrand Factor’s Role in the Prediction of Outcomes in Acute Ischemic Stroke. J. Clin. Med..

[25] Mital A., Prejzner W., Hellmann A. (2025). Management of acquired von Willebrand syndrome (AVWS). Acta Haematol. Pol..

[26] Wang H., Li D., Chen Y., Liu Z., Liu Y., Meng X., Fan H., Hou S. (2023). Shear-induced acquired von Willebrand syndrome: An accomplice of bleeding events in adults on extracorporeal membrane oxygenation support. Front. Cardiovasc. Med..

[27] Abu Khadija H., Alnees M., Ayyad O., Gandelman G., Abu Hamdeh N., Haim A., Hamdan Y., Cohen R., Najajra D., Kirzhner A. (2025). Pre-procedural abnormal von Willebrand factor function predicts clinical outcomes after Transcatheter Aortic Valve Implantation: A prospective cohort study. Front. Cardiovasc. Med..

[28] Roth N., Heidel C., Xu C., Hubauer U., Wallner S., Meindl C., Holzamer A., Hilker M., Creutzenberg M., Sossalla S. (2023). Restoration of von Willebrand factor after transcatheter aortic valve replacement—A possible cause for post-TAVR thrombocytopenia. Catheter. Cardiovasc. Interv..

[29] Grigorescu A.E., Anghel A., Koch C., Horhat F.G., Savescu D., Feier H. (2024). Von Willebrand Factor Dynamics in Patients with Aortic Stenosis Undergoing Surgical and Transcatheter Valve Replacement. Life.

[30] Avtaeva Y.N., Melnikov I.S., Komlev A.E. (2020). Shear stress induced activation of von Willebrand factor may predispose to gastrointestinal bleeding in syndrome of Heyde. Eur. Heart J..

[31] Zhang Y., Zhao D., Si X., Yue X., Chen J., Lu Y., Qiu P., Lu X., Yang X. (2024). Endograft-specific hemodynamics after endovascular aneurysm repair: A CFD analysis. Sci. Rep..

[32] Gniewek J., Krych S., Stępień-Słodkowska M., Adamczyk M., Hrapkowicz T., Kowalczyk P. (2026). Methodological and Pathophysiological Considerations in Obesity-Associated Thrombosis. Int. J. Mol. Sci..

[33] Okhota S., Melnikov I., Avtaeva Y., Kozlov S., Gabbasov Z. (2020). Shear Stress-Induced Activation of von Willebrand Factor and Cardiovascular Pathology. Int. J. Mol. Sci..

[34] Fuchizaki A., Yasui K., Hayashi T., Fujimura Y., Oyamada C., Ohnishi-Wada T., Hosokawa K., Shimogaki K., Kimura T., Hirayama F. (2024). Quantification of the contribution of individual coagulation factors to haemostasis using a microchip flow chamber system and reconstituted blood from deficient plasma. Vox Sang..

[35] Atari B., Ito T., Nagasato T., Ohnishi T., Hosokawa K., Yasuda T., Maruyama I., Kakihana Y. (2020). A modified microchip-based flow chamber system for evaluating thrombogenicity in patients with thrombocytopenia. Thromb. J..

[36] Penglong T., Boontanvansom A., Viboonjuntra P., Siripaitoon B. (2023). Reduced ADAMTS13 activity and high D-dimer levels are associated with thrombosis in patients with systemic lupus erythematosus. Blood Coagul. Fibrinolysis.

[37] Prasannan N., Dragunaite B., Subhan M., Thomas M., de Groot R., Singh D., Vanhoorelbeke K., Scully M. (2024). Peak ADAMTS13 activity to assess ADAMTS13 conformation and risk of relapse in immune-mediated thrombotic thrombocytopenic purpura. Blood.

[38] Papakonstantinou A., Kalmoukos P., Mpalaska A., Koravou E.-E., Gavriilaki E. (2024). ADAMTS13 in the New Era of TTP. Int. J. Mol. Sci..

[39] Chou E., Pirruccello J.P., Ellinor P.T., Lindsay M.E. (2023). Genetics and mechanisms of thoracic aortic disease. Nat. Rev. Cardiol..

[40] Cao W., Cao W., Zhang W., Zheng X., Zhang X. (2020). Factor VIII binding affects the mechanical unraveling of the A2 domain of von Willebrand factor. J. Thromb. Haemost..

[41] Lancellotti S. (2022). The von Willebrand Factor-ADAMTS-13 Axis: A Two-Faced Janus in Bleeding and Thrombosis. Bleeding Thromb. Vasc. Biol..

[42] Demoulin E., Jolou J., Schorer R., Walder B., Glessgen C., Huber C., Cikirikcioglu M. (2025). Floating Thrombus on the Ascending Aorta and/or Aortic Arch, to Operate or Not to Operate: Two Case Reports and a Literature Review. J. Cardiovasc. Dev. Dis..

[43] Ntalouka M.P., Makris T.K., Tsiligiridis K., Vasilellis A., Stamatelopoulos K.S., Kampolis C.F., Kakisis J.D., Kotsis T., Papadimitriou J.C. (2025). Evaluation of Coagulation Factors and Platelet Activation in Patients Undergoing Endovascular Aneurysm Repair of Para-renal and Thoraco-Abdominal Aortic Aneurysms. J. Clin. Med..

[44] Kobayashi T., Yamaguchi T., Nogami K., Shimizu T., Hoshiyama T., Kanazawa H., Hanatani S., Araki S., Usuku H., Nakamura T. (2020). Development and Assessment of a Total Thrombus Formation Analysis System-Based Bleeding Risk Model in Patients Undergoing Percutaneous Coronary Intervention. J. Thromb. Haemost..

[45] Capecchi E., Cortesi V., Raffaeli G., Picciolli I., Pesenti N., Fumagalli M., Cavallaro G., Ghirardello S., Francescato G. (2024). Evaluation of Platelet Function by Total Thrombus-Formation Analysis System (T-TAS^®^01) in Term and Preterm Infants and Its Relationship with Patent Ductus Arteriosus: A Prospective Observational Pilot Study. Blood Transfus..

[46] Krych S., Gniewek J., Kolbowicz M., Adamczyk M., Hrapkowicz T., Kowalczyk P. (2026). Oxidative Stress-Induced DNA Damage Response Pathways in Aortic Disease: Implications for Inflammation and Vascular Degeneration. Int. J. Mol. Sci..

[47] Korukonda S., Byers P.H., Kovuri P., Dhanekula A.S., Wilcox K.T., DeRoo S. (2025). Genotype–Aortic Phenotype Correlations in Marfan Syndrome: A Systematic Review and Meta-Analysis of Fibrillin-1 Variants. Heart.

[48] Pedroza A.J., Dalal A.R., Kim J., Duda M., Tognozzi E., Gilles C., Miller D.C., Fischbein M.P. (2025). Loeys-Dietz syndrome subtypes exhibit distinct clinical behavior and aortic cellular transcriptomic profiles. JTCVS Open.

[49] Nauth T., Philipp M., Renner S., Burkhalter M.D., Schüler H., Saygi C., Händler K., Siebels B., Busch A., Mair T. (2025). CDKL1 variants affecting ciliary formation predispose to thoracic aortic aneurysm and dissection. J. Clin. Investig..

[50] Dey S., Cheikhali R., Frishman W.H., Aronow W.S. (2024). Genetic Problems, Diagnosis, and Cardiovascular Manifestations of Loeys-Dietz Syndrome. Cardiol. Rev..

[51] Rios J.L., Magee G.A. (2025). Genetics of Thoracic and Thoracoabdominal Aortic Dissections and Aneurysms. Cardiol. Clin..

[52] Levy L.E., Zak M., Glotzbach J.P. (2024). Current Understanding of the Genetics of Thoracic Aortic Disease. Vessel Plus.

[53] Bobba C.M., Azarrafiy R., Spratt J.R., Hendrickson J., Martin T.D., Arnaoutakis G.J., Jeng E.I., Beaver T.M. (2023). A Highly Penetrant ACTA2 Mutation of Thoracic Aortic Disease. J. Cardiothorac. Surg..

[54] Tomida S., Ishima T., Sawaki D., Imai Y., Nagai R., Aizawa K. (2023). Multi-Omics of Familial Thoracic Aortic Aneurysm and Dissection: Calcium Transport Impairment Predisposes Aortas to Dissection. Int. J. Mol. Sci..

[55] Schwartzman W.E., Hujoel M.L.A., Channaoui N., Lee-Kim V., Loh P.-R., Gupta R.M. (2025). Interpreting MYH11 Copy Number Variation in Thoracic Aortic Aneurysm and Dissection: Insights from the Misannotation of Variants in Clinical Genetic Tests. JACC Case Rep..

[56] Gąsecka A., Kaczorowski R., Pomykała K., Kucharski T., Gajewska M., Siwik D., Karoń K., Małyszko M., Hunia J., Zimodro J.M. (2025). Effect of aspirin dosage on oxidative stress and platelet reactivity in patients undergoing coronary artery bypass grafting (APRICOT): Randomized controlled trial. Platelets.

[57] Yu S., Huang L., Ren J., Zhang X. (2024). Association of polymorphisms in FBN1, MYH11, and TGF-β signaling-related genes with susceptibility of sporadic thoracic aortic aneurysm and dissection in the Zhejiang Han population. Open Med..

[58] Zhou C., Zhao H., Jiang P., Sun L., Chang Y., Ma X., Du Z. (2023). Association of Gene Polymorphisms in ACTA2, MYH11, FBN1 and TGF-β Signaling with Susceptibility of DeBakey Type III Aortic Dissection. Case Rep. Genet..

[59] Monda E., Lioncino M., Verrillo F., Rubino M., Caiazza M., Mauriello A., Guarnaccia N., Fusco A., Cirillo A., Covino S. (2023). The Role of Genetic Testing in Patients with Heritable Thoracic Aortic Diseases. Diagnostics.

[60] Morisaki H. (2024). Hereditary Aortic Aneurysms and Dissections: Clinical Diagnosis and Genetic Testing. Ann. Vasc. Dis..

[61] Mahlmann A., Elzanaty N., Saleh M., Irqsusi M., Rastan A., Leip J.L., Behrendt C.-A., Ghazy T. (2024). Prevalence of Genetic Variants and Deep Phenotyping in Patients with Thoracic Aortic Aneurysm and Dissection: A Cross-Sectional Single-Centre Cohort Study. J. Clin. Med..

[62] Hiraoka D., Ishizaki J., Yamanouchi J., Honda T., Niiya T., Horimoto E., Horie K., Yamasaki H., Matsumoto T., Suemori K. (2024). Antiplatelet effects of hydroxychloroquine in patients with systemic lupus erythematosus evaluated by the total thrombus-formation analysis system (T-TAS). Lupus Sci. Med..

[63] Karczmarska-Wódzka A., Wszelaki P., Pstrągowski K., Sikora J. (2025). The Importance of Hemostasis on Long-Term Cardiovascular Outcomes in STEMI Patients—A Prospective Pilot Study. J. Clin. Med..

[64] Pfrepper C., von Ahsen N., Krüger S., Staudacher D.L., Meyer B., Heckmann S.M., Lubos E., Pötzsch B., Löschmann N., Messow C.M. (2024). Total Thrombus-Formation Analysis System in Patients with Peripheral Artery Disease Undergoing Revascularization: Assessment of DAPT Response. Platelets.

[65] Ueno T., Kamimura D., Miyagawa S., Okazaki T., Shirakura K., Kosuga K., Matsumiya G., Kawahito Y. (2020). Detection of Acquired von Willebrand Syndrome after Continuous-Flow Left Ventricular Assist Device Implantation Using the Total Thrombus-Formation Analysis System. ESC Heart Fail..

[66] Kontovazainitis C.-G., Gialamprinou D., Fleva A., Theodoridis T., Chatziioannidis I., Mitsiakou C., Banti A., Diamanti E., Mitsiakos G. (2025). Rotational Thromboelastometry (ROTEM) Hemostasis Profile in Pregnant Women with Preeclampsia and Their Offspring: An Observational Study. Diagnostics.

[67] Matsuo O., Ishii M., Kaikita K., Morinaga J., Miyamura F., Matsumoto S., Tsujita K., Nakamura K. (2023). Utility of the Total Thrombus-Formation Analysis System as a Tool for Evaluating Thrombogenicity and Monitoring Antithrombotic Therapy in Pediatric Fontan Patients. Pediatr. Cardiol..

[68] Calderon-Martinez E., Velasco W.V., Guo D., Hostetler E.H., Xun Z., Stephens S., Shalhub S., De Backer J., Ouzounian M., LeMaire S.A. (2025). Differences in Arterial Events in Vascular Ehlers–Danlos, Loeys–Dietz, and Marfan Syndrome. J. Am. Coll. Cardiol..

[69] Koefoed A.W., Huguenard A.L., Johnson G.W., Deych E., Guo D., Milewicz D.M., Braverman A.C. (2025). Characterization of Arterial Aneurysms in Loeys–Dietz Syndrome. J. Am. Coll. Cardiol..

[70] Li C., Chang C., Wang M., Bai Y., Zhang K., Geng J., Chen Q. (2025). Genome Wide DNA Methylation and Transcriptome Integration Analysis Reveals Potential Markers in Type A Aortic Dissection Pathogenesis. Sci. Rep..

[71] Rhee Y.H., Spin J.M., Tsao P.S. (2025). A Narrative Review of Recent Literature of Circulating Biomarkers of Abdominal Aortic Aneurysm and Their Translational Relevance. J. Vasc. Surg. Sci..

[72] Yan X., Li X., Qin Y.Y., Zheng J.H., Yong X., Li S.X., Lu Q.S. (2025). Trajectory of Platelet Changes Following Endovascular Abdominal Aortic Aneurysm Repair and Its Relationship with Postoperative Adverse Events. Zhonghua Yixue Zazhi.

[73] Gąsecka A., Błażejowska E., Konieczka A., Leśniewski M., Ostaszewska M., Łomiak M., Gajewska M., Rogula S., Szarpak Ł., Filipiak K.J. (2025). Branched Endovascular Aortic Aneurysm Repair Decreases Platelet Reactivity and Platelet-Rich Thrombus Formation—A Prospective Cohort Study. Platelets.

[74] Roth N., Heidel C., Xu C., Hubauer U., Wallner S., Meindl C., Holzamer A., Hilker M., Creutzenberg M., Sossalla S. (2022). The Impact of Bicuspid Aortic Valve Morphology on von Willebrand Factor Function in Patients with Severe Aortic Stenosis and Its Change After TAVI. Clin. Res. Cardiol..

[75] Yan S., Zhang Y., Shen C., Xiong J., Chen S., Ren Z. (2025). Clinical Factors Affecting Thrombosis of False Lumen After Thoracic Endovascular Aortic Repair in Patients with Complicated Stanford Type B Aortic Dissection. Chest Surg. Crit. Care.

[76] Carbone A., Monda E., Ferrara F., Franzese M., Bottino R., Russo V., Cirillo C., Rega S., Cittadini A., Pelliccia A. (2024). Aortic dimension in elite athletes: Updated systematic review and meta-analysis. Eur. J. Prev. Cardiol..

[77] Rodríguez-Palomares J.F., Oliveró R., Teixidó-Tura G. (2025). Understanding aortic enlargement in elite athletes: A physiological adaptation or pathological concern?. Eur. J. Prev. Cardiol..

[78] Limongelli G., Monda E., Lioncino M., Di Paolo F., Ferrara F., Vriz O., Calabrò P., Bossone E., Pelliccia A. (2023). Aortic Root Diameter in Highly-Trained Competitive Athletes: Reference Values According to Sport and Prevalence of Aortic Enlargement. Can. J. Cardiol..

[79] Tso J.V., Turner C.G., Liu C., Prabakaran G., Jackson M., Galante A., Gilson C.R., Clark C., Williams B.R., Quyyumi A.A. (2023). Longitudinal Aortic Root Dilatation in Collegiate American-Style Football Athletes. J. Am. Heart Assoc..

[80] Most A., Groesser V., Hoelscher S., Weber R., Akdogan-Gernandt E., Kraushaar L., Dörr O., Sedighi J., Keranov S., Husain-Syed F. (2024). Association of aortic root diameter and vascular function with an exaggerated blood pressure response to exercise among elite athletes. Clin. Res. Cardiol..

[81] Tang Q.-H., Yang H., Qin Z., Lin Q.-N., Hu M., Qin X., Chen J. (2025). Influencing Factors of False Lumen Thrombosis in Type B Aortic Dissection: A Single-Center Retrospective Study. Open Med..

[82] Armour C.H., Menichini C., Milinis K., Gibbs R.G.J., Xu X.Y. (2020). Location of Reentry Tears Affects False Lumen Thrombosis in Aortic Dissection Following TEVAR. J. Endovasc. Ther..

[83] Martinez M.W., Kim J.H., Shah A.B., Phelan D., Emery M.S., Wasfy M.M., Fernandez A.B., Bunch T.J., Dean P., Danielian A. (2021). Exercise-Induced Cardiovascular Adaptations and Approach to Exercise and Cardiovascular Disease: JACC State-of-the-Art Review. J. Am. Coll. Cardiol..

[84] Mammen E.F., Comp P.C., Gosselin R., Greenberg C., Hoots W.K., Kessler C.M., Larkin E.C., Liles D., Nugent D.J. (2024). PFA-100 System: A New Method for Assessment of Platelet Dysfunction. Semin. Thromb. Hemost..

[85] Kundu S.K., Heilmann E.J., Sio R., Garcia C., Davidson R.M., Ostgaard R.A. (2024). Description of an In Vitro Platelet Function Analyzer—PFA-100^®^. Semin. Thromb. Hemost..

[86] Fandaros M., Kwok C., Wolf Z., Labropoulos N., Yin W. (2024). Patient-Specific Numerical Simulations of Coronary Artery Hemodynamics and Biomechanics: A Pathway to Clinical Use. Cardiovasc. Eng. Technol..

[87] Schoenborn S., Pirola S., Woodruff M.A., Allenby M.C. (2024). Fluid-Structure Interaction Within Models of Patient-Specific Arteries: Computational Simulations and Experimental Validations. IEEE Rev. Biomed. Eng..

[88] Fernández M.A., Gerbeau J.F., Grandmont C. (2007). A projection semi-implicit scheme for the coupling of an elastic structure with an incompressible fluid. Int. J. Numer. Meth. Eng..

[89] Morris P.D., Narracott A., von Tengg-Kobligk H., Silva Soto D.A., Hsiao S., Lungu A., Evans P., Bressloff N.W., Lawford P.V., Hose D.R. (2016). Computational fluid dynamics modelling in cardiovascular medicine. Heart.

[90] Sotiropoulos F., Yang X. (2014). Immersed boundary methods for simulating fluid–structure interaction. Prog. Aerosp. Sci..

[91] Wang X., Carpenter H.J., Ghayesh M.H., Kotousov A., Zander A.C., Amabili M., Psaltis P.J. (2023). A review on the biomechanical behaviour of the aorta. J. Mech. Behav. Biomed. Mater..

[92] Sarantides P., Raptis A., Mathioulakis D., Moulakakis K., Kakisis J., Manopoulos C. (2024). Computational study of abdominal aortic aneurysm walls accounting for patient-specific non-uniform intraluminal thrombus thickness and distinct material models: A pre- and post-rupture case. Bioengineering.

[93] Jamshidian M., Wittek A., Sekhavat S., Miller K. (2025). Kinematics of abdominal aortic aneurysms. J. Biomech..

[94] Catalano C., Crascì F., Puleo S., Scuoppo R., Pasta S., Raffa G.M. (2025). Computational fluid dynamics in cardiac surgery and perfusion: A review. Perfusion.

[95] Reiter G., Reiter C., Ovcina I., Fuchsjäger M., Reiter U. (2025). Four-dimensional Flow MRI for a Dynamic Perspective on the Heart and Adjacent Great Vessels. Radiology.

[96] Power G., Ferreira-Santos L., Martinez-Lemus L.A., Padilla J. (2024). Integrating molecular and cellular components of endothelial shear stress mechanotransduction. Am. J. Physiol. Heart Circ. Physiol..

[97] Zhou M., Yu Y., Chen R., Liu X., Hu Y., Ma Z., Gao L., Jian W., Wang L. (2023). Wall shear stress and its role in atherosclerosis. Front. Cardiovasc. Med..

[98] Mokhtari A., Corso P., Jung B., Ferrari L., Zheng S., Obrist D. (2025). Comparison of 4D flow MRI and computational fluid dynamics in carotid models with different stenosis levels. Comput. Biol. Med..

[99] Loly V.T.R., Cintra A., Ramirez-Velandia F., Ogilvy C.S., Mensah E.O., de Sá Brasil Lima J., Nucci M.P., Baccin C.E., Gamarra L.F. (2025). Computational Fluid Dynamics Approaches for Analyzing Rupture and Growth of Intracranial Aneurysms: A Systematic Review. Biomedicines.

